# Supplementary material for: Tuning the reactivity of carbon surfaces with oxygen-containing functional groups
Source: Nat Commun. 2023 Apr 21;14:2293. doi: 10.1038/s41467-023-37962-3 (PMC10121666; doi:10.1038/s41467-023-37962-3)
Supplement: Supplementary file 1 — Supplementary Information [file 41467_2023_37962_MOESM1_ESM.pdf]

Supplementary Information

Tuning the reactivity of carbon surfaces with oxygen-containing functional groups

Jiahua Zhou,[1,2†] Piaoping Yang,[1,2†] Pavel A. Kots,[1] Maximilian Cohen,[1] Ying Chen,[3] Caitlin M. Quinn,[4] Matheus Dorneles de Mello,[2,5] J. Anibal Boscoboinik,[2,5] Wendy J. Shaw,[3] Stavros Caratzoulas,[2] Weiqing Zheng,[2*] Dionisios G. Vlachos[1,2*]

[1] Department of Chemical and Biomolecular Engineering, University of Delaware, Newark, DE 19716, USA

[2] Catalysis Center for Energy Innovation, University of Delaware, Newark, DE 19716, USA

[3]Pacific Northwest National Laboratory, Richland, WA 99352, USA

[4]Department of Chemistry and Biochemistry, University of Delaware, Newark, DE 19716, USA

[5]Center for Functional Nanomaterials, Brookhaven National Laboratory, Upton, NY 11973, USA

[†]These authors contributed equally to the work

*Corresponding authors: weiqing@udel.edu, vlachos@udel.edu

Table of Contents

Supplementary Figure 1……………………………………………………………………..4

Supplementary Table 1..……………………………………………………………………..5

Supplementary Figure 2……………………………………………………………………..6

Supplementary Table 2………………………………………………………………………7

Supplementary Figure 3……………………………………………………………………..8

Supplementary Figure 4……………………………………………………………………..9

Supplementary Note 1……………………………………………………………………...10

Supplementary Table 3……………………………………………………………………..10

Supplementary Table 4. ……………………………………………………………………...11

Supplementary Figure 5…………………………………………………………………....12

Supplementary Table 5……………………………………………………………………..13

Supplementary Table 6……………………………………………………………………..13

Supplementary Table 7……………………………………………………………………..14

Supplementary Table 8……………………………………………………………………..14

Supplementary Table 9……………………………………………………………………..15

Supplementary Table 10……………………………...………………………………………..15

Supplementary Table 11…………………………………………………………….………..16

Supplementary Table 12……………………………………………………………………16

Supplementary Table 13……………………………………………………………………17

Supplementary Note 2……………………………………………………………………...18

Supplementary Figure 6…………………………………………………………………....18

Supplementary Table 14……………………………………………………………………19

Supplementary Note 3……………………………………………………………………...20

Supplementary Table 15……………………………………………………………………20

Supplementary Figure 7……………………………………………………………………21

Supplementary Table 16……………………………………………………………………22

Supplementary Table 17……………………………………………………………………23

Supplementary Table 18……………………………………………………………………24

Supplementary Table 19……………………………………………………………………25

Supplementary Figure 8……………………………………………………………………26

Supplementary Figure 9……………………………………………………………………27

Supplementary Figure 10…………………………………………………………………..28

Supplementary Figure 11……………………………………………………………..29
Supplementary Figure 12..…………………………………………………………30
Supplementary Figure 13..…………………………………………………………31
Supplementary Figure 14..…………………………………………………………32
Supplementary Figure 15..…………………………………………………………33
Supplementary Figure 16..…………………………………………………………34
Supplementary Figure 17..…………………………………………………………35
Supplementary Note 4……………………………………………………………...36
Supplementary Table 20……………………………………………………………36
Supplementary Table 21……………………………………………………………36
Supplementary Figure 18..…………………………………………………………37
Supplementary Figure 19..…………………………………………………………38
Supplementary Figure 20……………………………………………………………..39
Supplementary Figure 21……………………………………………………………..40
Supplementary Figure 22……………………………………………………………..41
Supplementary Note 5……………………………………………………………...42
Supplementary Figure 23……………………………………………………………..42
Supplementary Figure 24……………………………………………………………..43
Supplementary References..…………………………………………………………..44

Figure S1.

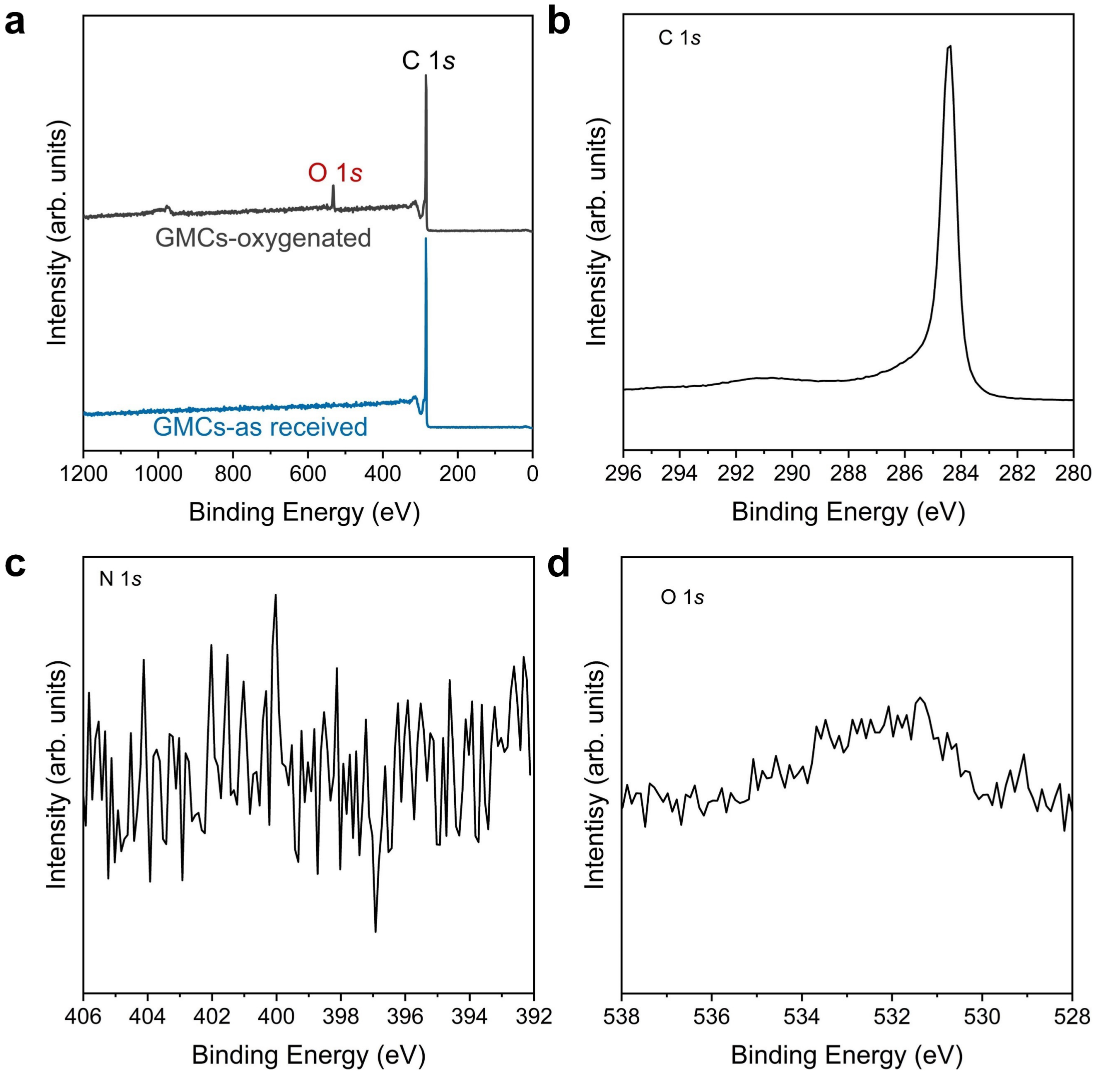

Figure S1. XPS spectra of GMCs-as received. a survey spectrum of GMCs-as received and GMCs-oxygenated samples. **b**, **c**, **d** C 1*s*, O 1*s*, N 1*s* core-lever spectra of GMCs-as received.

Table S1. Elemental composition of pristine and functionalized GMCs at various treatments.

Carbon samples	Oxidation duration (h)	Annealing temperature (°C)	C[a] (wt%)	O[a] (wt%)	C[b] (wt%)	O[b] (wt%)	Others[b] (wt%)
GMCs-as received	0	200	99.36	0.64	-	-	-
GMCs-ox (48 h)-200 °C	48	200	91.71	8.29	91.13	8.21	0.66
GMCs-ox (48 h)-400 °C	48	400	93.49	6.51	93.75	5.74	0.52
GMCs-ox (48 h)-600 °C	48	600	95.41	4.59	95.54	4.28	0.18
GMCs-ox (48 h)-800 °C	48	800	97.01	2.99	97.94	1.22	0.84
GMCs-ox (24 h)-200 °C	24	200	93.51	6.49	93.01	6.08	0.91
GMCs-ox (24 h)-400 °C	24	400	95.28	4.72	94.53	4.51	0.97
GMCs-ox (24 h)-600 °C	24	600	97.02	2.98	96.91	2.74	0.35
GMCs-ox (24 h)-800 °C	24	800	98.18	1.82	98.27	1.70	0.034
GMCs-ox (12 h)-200 °C	12	200	94.88	5.12	94.49	4.84	0.67
GMCs-ox (12 h)-200 °C	12	400	96.52	3.48	96.17	3.26	0.57
GMCs-ox (12 h)-200 °C	12	600	97.42	2.58	96.94	2.07	0.99
GMCs-ox (12 h)-200 °C	12	800	98.37	1.63	97.15	1.52	1.33
GMCs-ox (6 h)-200 °C	6	200	96.00	3.99	96.04	3.75	0.21
GMCs-ox (6 h)-200 °C	6	400	96.89	3.11	97.89	1.90	0.208
GMCs-ox (6 h)-200 °C	6	600	97.86	2.14	98.01	1.52	0.471
GMCs-ox (6 h)-200 °C	6	800	98.59	1.41	98.56	1.33	0.108

[a] Surface elemental composition determined by XPS. Difference to 100%, sensitivity factors: C 1*s* 0.278, O 1*s* 0.78; from the Kratos library. [b] Bulk elemental composition determined by CHNS measurement.

Figure S2.

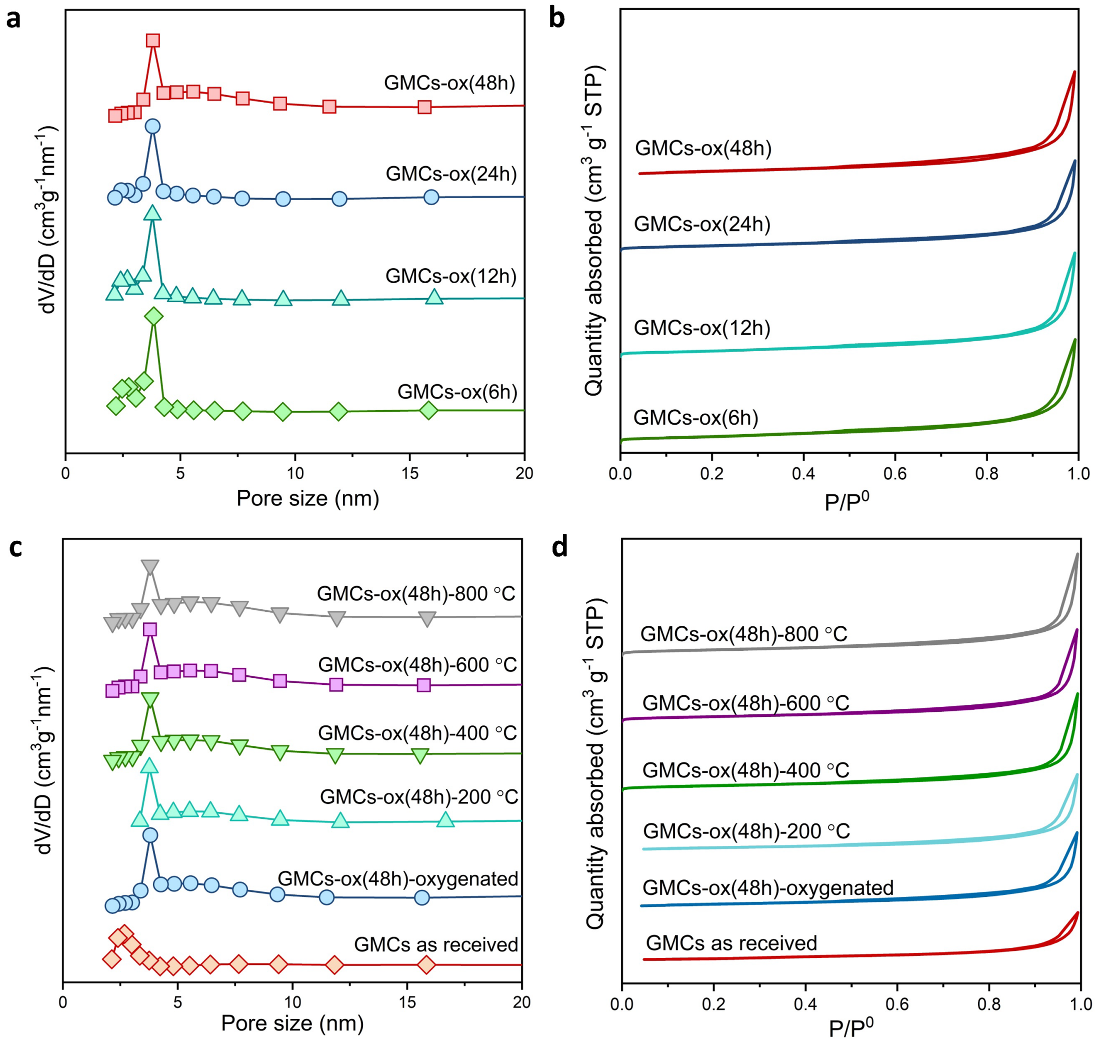

Figure S2. N_2 adsorption/desorption isotherms at -196 °C of samples at various treatments. **a**, **c** BJH pore size distribution. **b**, **d** N_2 adsorption-desorption isotherms.

Table S2. Textural and physical properties of the oxygenated carbon materials.

Samples of different oxidation time

Carbon	S_{BET}[a] (m^2/g)	D_{pore}[a] (nm)	V_{pore}[a] (cm^3/g)
GMCs	74.4	2.67	0.34
GMCs-ox(48h)-dry	101.3	3.81	0.52
GMCs-ox(24h)-dry	82.0	3.79	0.44
GMCs-ox(12h)-dry	89.2	3.79	0.51
GMCs-ox(6h)-dry	97.3	3.86	0.51

[a]*Determined by BET.*

Samples of different annealing temperature

Carbon	S_{BET}[a] (m^2/g)	D_{pore}[a] (nm)	V_{pore}[a] (cm^3/g)
GMCs	74.4	2.67	0.34
GMCs-ox(48h)	101.3	3.81	0.52
GMCs-ox(48h)-200°C	94.1	3.75	0.52
GMCs-ox(48h)-400°C	102.7	3.84	0.65
GMCs-ox(48h)-600°C	105.9	3.81	0.62
GMCs-ox(48h)-800°C	110.0	3.81	0.68

[a]*Determined by BET.*

The surface area, pore size, and volume are nearly the same under various treatments, indicating that most of the OCFGs are on the outer surface of the carbon. Concentrated nitric acid changes the carbon structure features.

Figure S3.

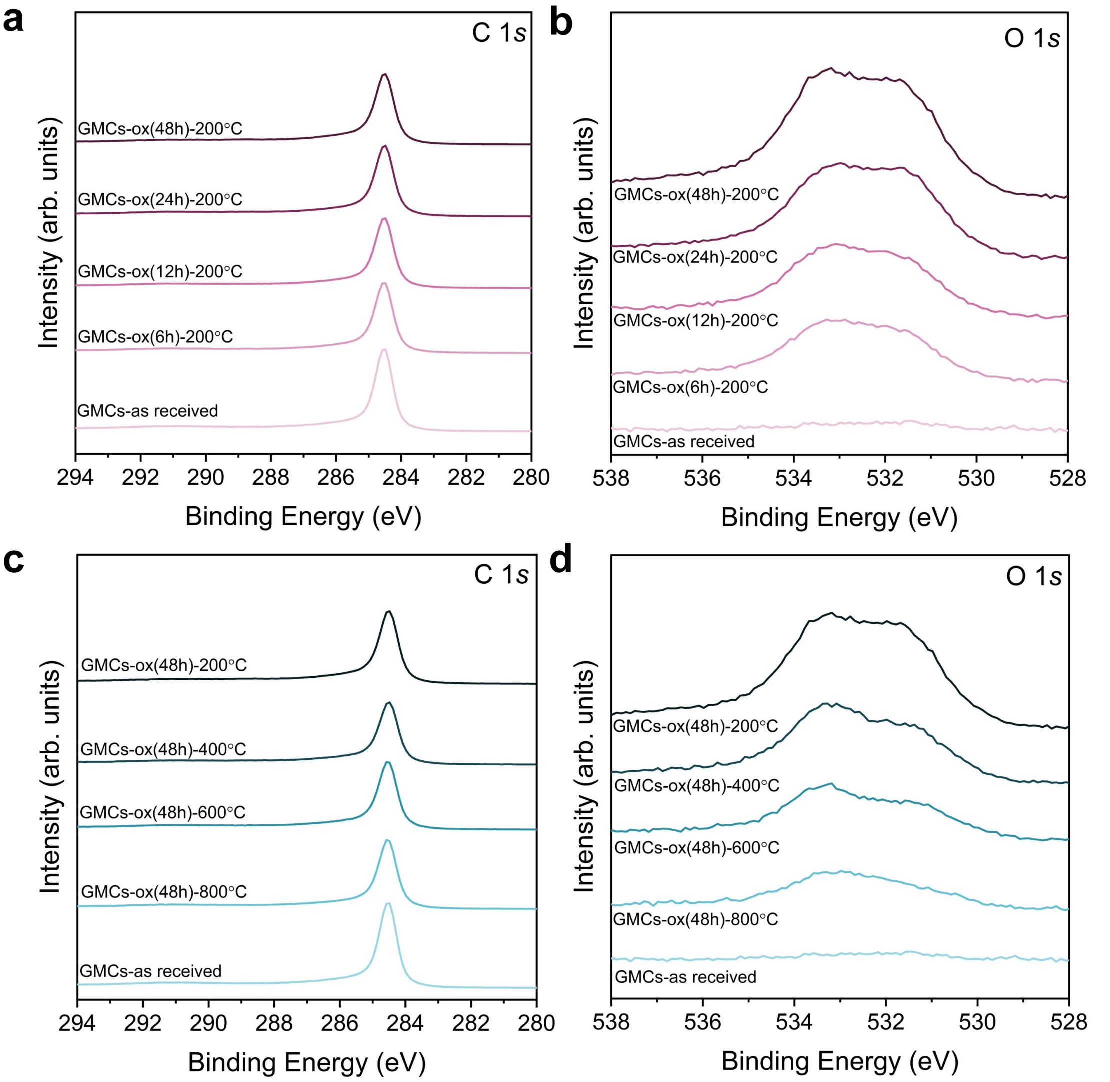

Figure S3. C 1*s* and O 1*s* XPS spectra. Samples at various **a**, **b** oxidation times, and **c**, **d** annealing temperatures.

Figure S4.

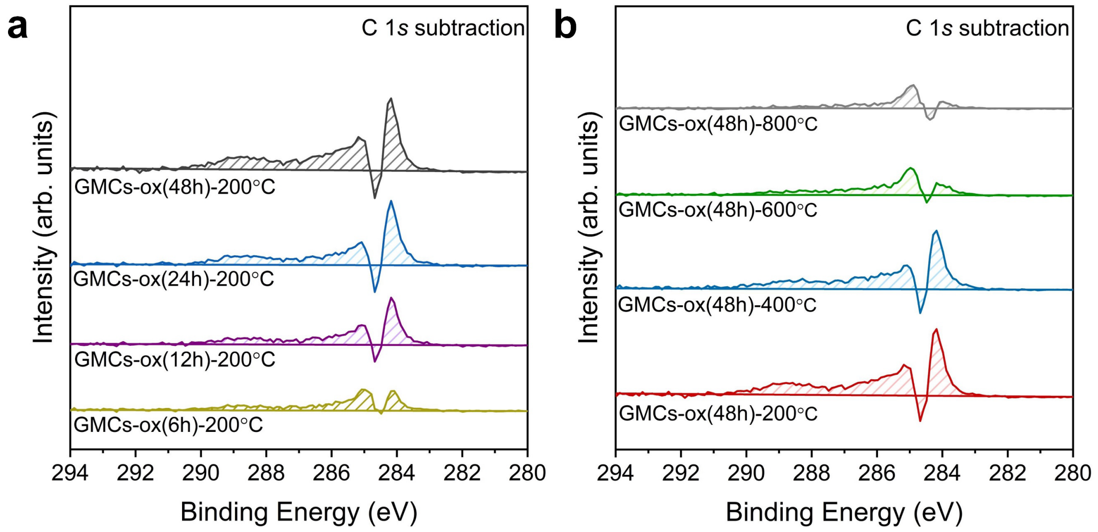

Figure S4. XPS difference spectra (C 1*s* subtraction from the as received GMCs). Samples at various **a** oxidation times and **b** annealing temperatures.

Supplementary Note 1

C 1*s* and O 1*s* fitting parameters

It was reported that the C 1*s* features could be deconvoluted into multiple peaks representing chemical states of carbon trigonal geometry.[1,2] Graphitized carbon has a unique asymmetric peak near 284.4 eV interpreted as sp^2 carbon (C=C).[3] Specifically, the FWHM of sp^2 is narrower than other species due to the highly graphitized C=C structure and the monochromatic aluminum Kα X-ray source (300-400 nm) we use for the measurement. For the rest of the species, the FWHM is the same and is constrained to 0.5-1 eV but can be broader than sp^2. The symmetric peak at approximately 284.8 eV is assigned to sp^3 carbon (C-C).[4] The contribution of oxygen in the C 1*s* peak higher than 285 eV corresponds to multiple OCFGs. The C-O single-bonded groups at lower binding energy around 285.5 eV are assigned to the alcoholic, phenolic (hydroxyl), and ethers groups. The C-O at higher binding energy near 286.2 eV is attributed to keto-enolic equilibria or furans. Other highly oxidized peaks at 287.4 eV are assigned to C=O double bonds like ketone and quinones. The binding energy at around 288.7 eV is assigned to -COO (carboxylic or lactones).[5] Satellite features, which are also known as shake-up, are due to π-π* (>290 eV) transitions in which the electrons in the highest occupied molecular orbitals are promoted to the lowest unoccupied molecular orbitals.[6,7] To make the analysis more accurate, we combine the analysis of O 1*s* and C 1*s* spectra. Based on the linewidth of the O 1*s* spectra and the recommendation of fitting parameters for the graphitic carbon materials,[8] fitting with 5 to 6 peaks with an average FWHM of at least 1.0-2.0 eV per peak is feasible for the graphitized carbon material. The detailed peak assignment is summarized in Table S3 and Table S4.

These fittings were consistently used for all spectra, and the residual standard deviation (STD) was below 5% (closer to 1%). The C 1*s* and O 1*s* spectra peak assignments were based on literature.[6,8,13] Due to the high complexity of the O 1*s* spectrum, the core-level O 1*s* spectra fitting was performed on the O 1*s* difference spectra (Fig. 2b) obtained from the samples annealed at different temperatures. This allows one to precisely distinguish the O 1*s* spectrum of thermally unstable from thermally more stable species. Care was taken that all the fittings were self-consistent and could be compared quantitatively.

Table S3. C 1*s* peak assignment.

Peak number	Peak position (eV)	Assignment[2,6,8,9]
C1	284.4	C=C, sp^2 carbon
C2	284.8	C-C, sp^3 carbon
C3	285.5	C-O (Aliphatic C-H, C-O in alcohols, phenols, ethers)
C4	286.2	C=C-O (in keto-enolic equilibria or furans)
C5	287.4	C=O (quinones, ketones and aldehydes, pyrone, and carbonyl)
C6	288.7	O-C=O (COOH, COOR in carboxyl, lactones or esters, carboxylic anhydrides)
C7, C8	>290	π-π^* satellite

Table S4. O 1*s* peak assignment.

Peak number	Peak position (eV)	Assignment[8,9]
O1	530.5	C=O (in quinones)
O2	531.2	C=O (in ketones, aldehydes, lactones, esters, anhydrides, pyrone, isolated carbonyl)
O3	531.9	C-O-C (in aromatics (furan) or keto-enolic tautomers)
O4	532.7	C-OH (in phenolic or aliphatic alcohols) or chemisorbed H_2O
O5	533.5	C-O-C (in ethers, esters, anhydrides, lactones)
O6	534.2	C-OH (in carboxylic acid) or chemisorbed H_2O
O7	535~536	Gas-phase H_2O

Figure S5.

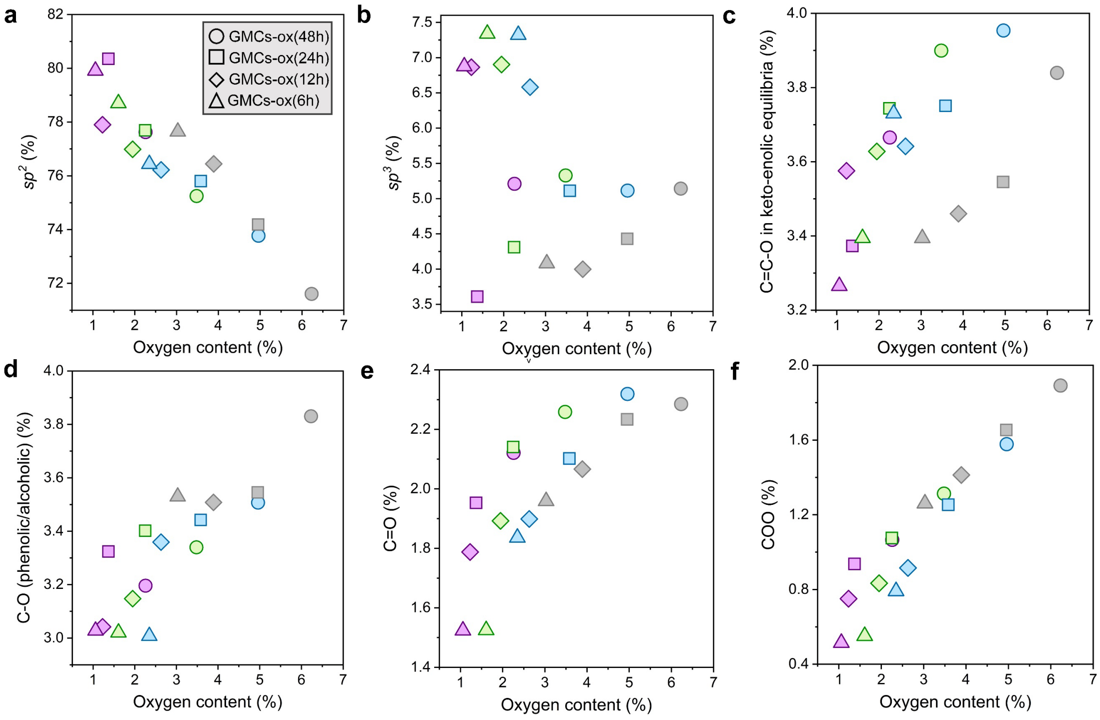

Figure S5. Ex situ XPS characterization of the oxygenated-GMCs samples treated for different oxidation times and at various annealing temperatures with the relative content of carbon structure and OCFGs from the C 1*s* spectra. **a** sp^2-hybridization carbon, **b** sp^3-hybridization carbon, **c** C-O from phenolic/alcoholic group, **d** C=C-O in keto-enolic equilibria group, **e** C=O group, **f** COO from carboxylic/lactone group. Black 200 °C, Blue 400 °C, Green 600 °C, Purple 800 °C.

Monte Carlo error analysis of C 1*s* and O 1*s* peak fitting models

To estimate the errors in the fitting model and the uncertainties of the fitting parameters, we did a comprehensive Monte Carlo error analysis of our fitting models. Monte Carlo error analysis for peak models provides an error estimate for the precision with which the optimization process determines fitting parameters given the expected noise in the data. The error analysis was performed by CASA XPS (2.3.22 PR1.0) software.

Table S5. Standard deviation (STD DEV) of peak positions from the C 1*s* fitting.

Component name[a]	Peak position (eV)	% STD DEV
^{C1} sp^2	284.393	1.37E-03
^{C2} sp^3	284.793	1.37E-03
^{C3} C-O (phenolic/alcoholic, etc.)	285.493	1.37E-03
^{C4} C-O (furan/keto-enolic, etc.)	286.193	1.37E-03
^{C5} C=O	287.393	1.37E-03
^{C6} COO (COOH/COOR)	288.693	1.37E-03
^{C7} π-π^* satellite	290	8.32E-03
^{C8} π-π^* satellite	291.318	2.96E-03

a. Fitting parameter of sample GMCs-ox(48h)-200 °C

Table S6. Standard deviation (STD DEV) of the C 1*s* fitting peak FWHM.

Component name[a]	FWHM	% STD DEV
^{C1} sp^2	0.6603	2.72E-03
^{C2} sp^3	1	1.91E-02
^{C3} C-O (phenolic/alcoholic, etc.)	1	1.91E-02
^{C4} C-O (furan/keto-enolic, etc.)	1	1.91E-02
^{C5} C=O	1	1.91E-02
^{C6} COO (COOH/COOR)	1	1.91E-02
^{C7} π-π^* satellite	1.787	6.71E-02
^{C8} π-π^* satellite	1.787	6.71E-02

a. Fitting parameter of sample GMCs-ox(48h)-200 °C

For C 1*s* spectra fittings, the STD DEV of the peak position/FWHM of different species in each sample is on the same order of magnitude and below (or close) 5% (closer to 1%), suggesting the fitting parameters and the fitting model is reasonable and stable.

Table S7. Monte Carlo error matrix of peak position from C 1*s* fitting.

Component Name[a]	C1 *sp²*	C2 *sp³*	C3 C-O (phenolic/ alcoholic, etc.)	C4 C-O (furan/keto-enolic, etc.)	C5 C=O	C6 COO (COOH/ COOR)	C7 π-π* satellite	C8 π-π* satellite
C1 *sp²*	1.88E-06	1.88E-06	1.88E-06	1.88E-06	1.88E-06	1.88E-06	5.99E-07	-4.21E-07
C2 *sp³*	1.88E-06	1.88E-06	1.88E-06	1.88E-06	1.88E-06	1.88E-06	5.99E-07	-4.21E-07
C3 C-O (phenolic/ alcoholic, etc.)	1.88E-06	1.88E-06	1.88E-06	1.88E-06	1.88E-06	1.88E-06	5.99E-07	-4.21E-07
C4 C-O (furan/keto-enolic, etc.)	1.88E-06	1.88E-06	1.88E-06	1.88E-06	1.88E-06	1.88E-06	5.99E-07	-4.21E-07
C5 C=O	1.88E-06	1.88E-06	1.88E-06	1.88E-06	1.88E-06	1.88E-06	5.99E-07	-4.21E-07
C6 COO (COOH/ COOR)	1.88E-06	1.88E-06	1.88E-06	1.88E-06	1.88E-06	1.88E-06	5.99E-07	-4.21E-07
C7 π-π* satellite	5.99E-07	5.99E-07	5.99E-07	5.99E-07	5.99E-07	5.99E-07	6.92E-05	-2.02E-06
C8 π-π* satellite	-4.21E-07	-4.21E-07	-4.21E-07	-4.21E-07	-4.21E-07	-4.21E-07	-2.02E-06	8.79E-06

a. Fitting parameter of sample GMCs-ox(48h)-200 °C

Table S8. Monte Carlo error matrix of FWHM from C 1*s* fitting.

Component Name[a]	C1 *sp²*	C2 *sp³*	C3 C-O (phenolic/ alcoholic, etc.)	C4 C-O (furan/keto-enolic, etc.)	C5 C=O	C6 COO (COOH/ COOR)	C7 π-π* satellite	C8 π-π* satellite
C1 *sp²*	7.41E-06	-5.46E-06	-5.46E-06	-5.46E-06	-5.46E-06	-5.46E-06	-6.52E-06	-6.52E-06
C2 *sp³*	-5.46E-06	3.66E-04	3.66E-04	3.66E-04	3.66E-04	3.66E-04	2.38E-04	2.38E-04
C3 C-O (phenolic/ alcoholic, etc.)	-5.46E-06	3.66E-04	3.66E-04	3.66E-04	3.66E-04	3.66E-04	2.38E-04	2.38E-04
C4 C-O (furan/keto-enolic, etc.)	-5.46E-06	3.66E-04	3.66E-04	3.66E-04	3.66E-04	3.66E-04	2.38E-04	2.38E-04
C5 C=O	-5.46E-06	3.66E-04	3.66E-04	3.66E-04	3.66E-04	3.66E-04	2.38E-04	2.38E-04
C6 COO (COOH/ COOR)	-5.46E-06	3.66E-04	3.66E-04	3.66E-04	3.66E-04	3.66E-04	2.38E-04	2.38E-04
C7 π-π* satellite	-6.52E-06	2.38E-04	2.38E-04	2.38E-04	2.38E-04	2.38E-04	4.50E-03	4.50E-03
C8 π-π* satellite	-6.52E-06	2.38E-04	2.38E-04	2.38E-04	2.38E-04	2.38E-04	4.50E-03	4.50E-03

a. Fitting parameter of sample GMCs-ox(48h)-200 °C

For C 1*s* spectra fittings, the Monte Carlo error matrix of the peak position/FWHM between different species in each sample is almost on the same order of magnitude and below 1%, further indicating the fitting parameters and the fitting model is reasonable and stable.

Table S9. Standard deviation (STD DEV) of peak positions in the O 1*s* fitting.

Component name[a]	Peak position (eV)	% STD DEV
O1 C=O	530.6	5.99E-03
O2 C=O	531.3	5.99E-03
O3 C-O-C	532	5.99E-03
O4 C-OH	532.8	5.99E-03
O5 C-O-C	533.4	5.99E-03
O6 C-OH	534.3	5.99E-03
O7 H_2O	536.1	5.99E-03

a. Fitting parameter of sample GMCs-ox(48h)-200 °C

Table S10. Standard deviation (STD DEV) of FWHM in the O 1*s* fitting.

Component name[a]	FWHM	% STD DEV
O1 C=O	1.8257	3.34E-02
O2 C=O	1.8257	3.34E-02
O3 C-O-C	1.8257	3.34E-02
O4 C-OH	1.8257	3.34E-02
O5 C-O-C	1.8257	3.34E-02
O6 C-OH	1.8257	3.34E-02
O7 H_2O	1.8257	3.34E-02

a. Fitting parameter of sample GMCs-ox(48h)-200 °C

For O 1*s* spectra fittings, the STD DEV of the peak position/FWHM of different species in each sample is on the same order of magnitude and below (or close) 5% (closer to 1%), suggesting the fitting parameters and fitting model is reasonable and stable.

Table S11. Monte Carlo error matrix of peak position from O 1*s* fitting.

Component Name[a]	O1 C=O	O2 C=O	O3 C-O-C	O4 C-OH	O5 C-O-C	O6 C-OH	O7 H_2O
O1 C=O	3.59E-05	3.59E-05	3.59E-05	3.59E-05	3.59E-05	3.59E-05	3.59E-05
O2 C=O	3.59E-05	3.59E-05	3.59E-05	3.59E-05	3.59E-05	3.59E-05	3.59E-05
O3 C-O-C	3.59E-05	3.59E-05	3.59E-05	3.59E-05	3.59E-05	3.59E-05	3.59E-05
O4 C-OH	3.59E-05	3.59E-05	3.59E-05	3.59E-05	3.59E-05	3.59E-05	3.59E-05
O5 C-O-C	3.59E-05	3.59E-05	3.59E-05	3.59E-05	3.59E-05	3.59E-05	3.59E-05
O6 C-OH	3.59E-05	3.59E-05	3.59E-05	3.59E-05	3.59E-05	3.59E-05	3.59E-05
O7 H_2O	3.59E-05	3.59E-05	3.59E-05	3.59E-05	3.59E-05	3.59E-05	3.59E-05

a. Fitting parameter of sample GMCs-ox(48h)-200 °C

Table S12. Monte Carlo error matrix of FWHM from O 1*s* fitting.

Component Name[a]	O1 C=O	O2 C=O	O3 C-O-C	O4 C-OH	O5 C-O-C	O6 C-OH	O7 H_2O
O1 C=O	1.12E-03	1.12E-03	1.12E-03	1.12E-03	1.12E-03	1.12E-03	1.12E-03
O2 C=O	1.12E-03	1.12E-03	1.12E-03	1.12E-03	1.12E-03	1.12E-03	1.12E-03
O3 C-O-C	1.12E-03	1.12E-03	1.12E-03	1.12E-03	1.12E-03	1.12E-03	1.12E-03
O4 C-OH	1.12E-03	1.12E-03	1.12E-03	1.12E-03	1.12E-03	1.12E-03	1.12E-03
O5 C-O-C	1.12E-03	1.12E-03	1.12E-03	1.12E-03	1.12E-03	1.12E-03	1.12E-03
O6 C-OH	1.12E-03	1.12E-03	1.12E-03	1.12E-03	1.12E-03	1.12E-03	1.12E-03
O7 H_2O	1.12E-03	1.12E-03	1.12E-03	1.12E-03	1.12E-03	1.12E-03	1.12E-03

a. Fitting parameter of sample GMCs-ox(48h)-200 °C

For O 1*s* spectra fittings, the Monte Carlo error matrix of the peak position/FWHM between different species in each sample is on the same order of magnitude and below 1%, further indicating the fitting parameters and the fitting model is reasonable and stable.

Table S13. Residual STD of the fitting results of all oxidized samples.

Samples	Residual STD of C 1*s* fitting (%)	Residual STD of O 1*s* fitting (%)
GMCs-ox(48h)-200 °C	3.887	1.067
GMCs-ox(48h)-400 °C	3.345	1.082
GMCs-ox(48h)-600 °C	3.72	1.273
GMCs-ox(48h)-800 °C	3.385	0.968
GMCs-ox(24h)-200 °C	4.686	0.9205
GMCs-ox(24h)-400 °C	4.771	1.516
GMCs-ox(24h)-600 °C	5.016	1.156
GMCs-ox(24h)-800 °C	4.65	0.95
GMCs-ox(12h)-200 °C	4.381	1.061
GMCs-ox(12h)-400 °C	2.906	1.019
GMCs-ox(12h)-600 °C	3.341	1.013
GMCs-ox(12h)-800 °C	3.213	0.997
GMCs-ox(6h)-200 °C	4.34	1.1
GMCs-ox(6h)-400 °C	2.967	1.031
GMCs-ox(6h)-600 °C	3.437	1.133
GMCs-ox(6h)-800 °C	3.062	0.99

The overall fitting residual STD of C 1*s* spectra for each sample is lower than 5%, which is acceptable, and the error might result from the line shape of sp^2 carbon which is unavoidable.

The overall fitting residual STD of O 1*s* spectra for each sample is lower than 5% (closer to 1%), indicating a stable fitting model.

Supplementary Note 2

Surface OFCGs concentration correction

The photoelectrons irradiated in XPS cannot pass through more than about 4 nm in depth,[10] therefore, the oxygen content from the XPS is the surface component. The CHNS (Table S16) was conducted to quantify the oxygen content from the bulk, and correct the OFCGs concentration with the equations (equation 1 and 2):

$$O_{\text{surface}} = \frac{O_s}{O_b + C_b} = \frac{\frac{O_s}{C_b}}{\frac{O_b}{C_b}+1} = \frac{\frac{O_s}{C_s}}{\frac{O_b}{C_b}+1} \times \frac{C_s}{C_b} \quad (1)$$

$$C_{\text{surface}} = \frac{C_s}{O_b + C_b} = \frac{\frac{C_s}{O_b}}{\frac{C_b}{O_b}+1} = \frac{\frac{C_s}{O_s}}{\frac{C_b}{O_b}+1} \times \frac{O_s}{O_b} \quad (2)$$

Where O_s/C_s is determined by XPS, and O_b/C_b is calculated by CHNS measurements. The unit of O_{surface} and C_{surface} is (g_C/g_{cat}) means the concentration of O and C in the surface layer that XPS can detect from the outer surface. Five points were eliminated from the 16 points due to the large error bar of the CHNS result (Table S1, Fig. S6). Finally, the corrected O and C concentrations (Table S14) of 11 points are applied in the following data analysis (including PLS analysis and TOF estimations).

Figure S6

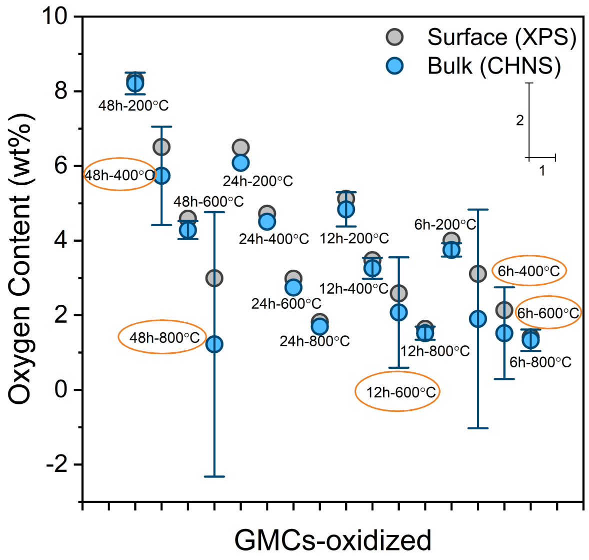

Figure S6. Comparison of oxygen content between the surface and bulk. Oxygen content calculated by XPS and CHNS, and error bar. Error bars represent the standard deviation from triplicates.

Table S14. Corrected elemental composition of the oxidized GMCs samples.

Samples	Surface content (corrected) (wt%)	
	C	O
GMCs-ox(48h)-200 °C	92.32	8.35
GMCs-ox(48h)-600 °C	95.59	4.60
GMCs-ox(24h)-200 °C	94.36	6.55
GMCs-ox(24h)-400 °C	96.21	4.76
GMCs-ox(24h)-600 °C	97.36	2.99
GMCs-ox(24h)-800 °C	98.22	1.82
GMCs-ox(12h)-200 °C	95.52	5.15
GMCs-ox(12h)-400 °C	97.08	3.50
GMCs-ox(12h)-800 °C	99.69	1.66
GMCs-ox(6h)-200 °C	96.20	4.01
GMCs-ox(6h)-800 °C	98.70	1.41

Supplementary Note 3

Quantification of individual intrinsic oxygen functional groups (Gi)

To determine the concentration of intrinsic **Gi** (mol_{Gi}/g_{cat}) (Figure S7) from OCFGs (mol_{Cj}/g_{cat} or mol_{Oj}/g_{cat}) obtained from the XPS spectra fitting and corrected by CHNS, we solve the balance equations shown below,

$$OCFG_j = \sum_i k_{ij} G_i \text{, j = 1 to 10; i = 1 to 10} \qquad (3)$$

where $k_{ij} = \dfrac{mol\ OCFG_j}{mol\ G_i}$; e.g., $k_{11} = 0$, $k_{31}=1$, $k_{71} = 2$.

The matrix of k_{ij} is shown in Table S15. To ensure a full rank matrix of k_{ij}, **G1** (the pyrone group) and **G8** (the lactol group) are removed since **G1** is a base group rarely generated and **G8** is unstable under typical conditions. We then solve for the values of **Gi** using eq. (1). The ordinary least square (OLS) regression was first employed but showed negative values of **Gi** which are not physically possible (Table S16). Hence, non-negative least square (NNLS) regression was used to obtain non-negative values of **Gi** (Table S17). However, the values of **G9** throughout the 11 samples are zero, indicating that **G9** does not exist in our samples. Thus, we removed **G9**. The R^2 values of NNLS regression are generally the same as those of the OLS regression, suggesting that it is feasible to have non-negative values (Table S18). Standardizing the initial dehydration rate and **Gi** (Table S17, resolved from the NNLS regression) was performed by z-score normalization (Table S19). We performed the data analysis using the Gi obtained from the NNLS regression (Table S17).

Table S15. Matrix of k_{ij}.

	G1 (i = 1)	G2 (i = 2)	G3 (i = 3)	G4 (i = 4)	G5 (i = 5)	G6 (i = 6)	G7 (i = 7)	G8 (i = 8)	G9 (i = 9)	G10 (i = 10)
OCFGs1 (C3, j = 1)	0	0	1	0	0	0	2	1	0	0
OCFGs2 (C4, j = 2)	0	0	0	0	0	2	0	0	0	0
OCFGs3 (C5, j = 3)	1	2	0	0	1	0	0	0	0	0
OCFGs4 (C6, j = 4)	0	0	0	1	0	0	0	1	1	2
OCFGs5 (O1, j = 5)	0	2	0	0	0	0	0	0	0	0
OCFGs6 (O2, j = 6)	1	0	0	1	1	0	0	1	1	2
OCFGs7 (O3, j = 7)	0	0	0	0	0	1	0	0	0	0
OCFGs8 (O4, j = 8)	0	0	1	0	0	0	0	1	0	0
OCFGs9 (O5, j = 9)	1	0	0	0	0	0	1	1	1	1
OCFGs10 (O6, j = 10)	0	0	0	1	0	0	0	0	0	0

G1: Pyrone group; **G2**: Quinone group; **G3**: Hydroxyl & Phenolic group; **G4**: Ar-carboxyl & R-carboxyl group; **G5**: Isolated carbonyl group; **G6**: Furan group; **G7**: Ether group; **G8**: Lactol group; **G9**: Lactone group; and **G10**: Anhydride group. The groups are depicted in Figure S7, Fig. 1a and Fig. 3g.

Figure S7 Oxygen-containing functional groups

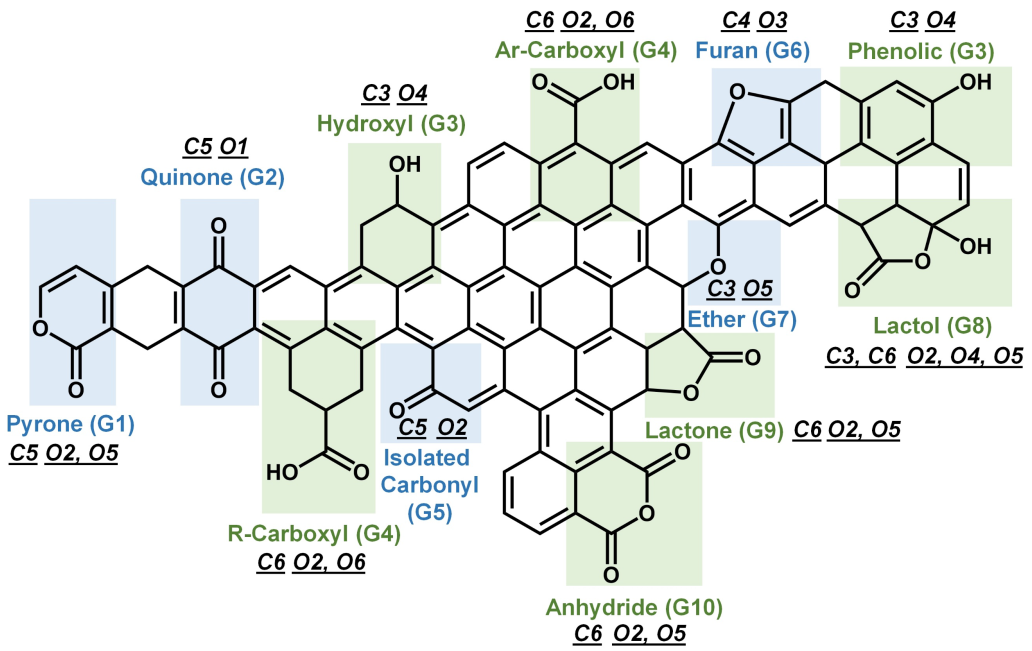

Figure S7. Oxygen-containing functional groups on carbon surfaces and the relative relationship between the OCFGs from the XPS fitting results (**C3-C6** (j = 1-4), **O1-O6** (j = 5-10)) and the individual oxygen functional species (**Gi**).

Table S16. Values of Gi from the ordinary least square (OLS) regression.

Samples	G2	G3	G4	G5	G6	G7	G10
1	2.32E-04	9.41E-04	2.13E-04	9.92E-04	1.37E-03	1.10E-03	4.57E-04
2	3.52E-04	2.21E-04	8.82E-05	6.55E-04	1.39E-03	1.22E-03	2.01E-04
3	3.00E-04	1.01E-03	3.42E-04	8.35E-04	1.27E-03	9.02E-04	2.48E-04
4	2.92E-04	4.40E-04	1.46E-04	7.04E-04	1.34E-03	1.16E-03	1.67E-04
5	3.72E-04	3.19E-04	2.13E-04	5.44E-04	1.33E-03	1.15E-03	-1.02E-05
6	3.00E-04	2.96E-04	2.77E-04	5.53E-04	1.20E-03	1.09E-03	-1.22E-04
7	2.71E-04	9.74E-04	3.75E-04	7.37E-04	1.22E-03	8.90E-04	1.06E-04
8	2.32E-04	3.71E-04	1.93E-04	7.36E-04	1.26E-03	1.14E-03	2.05E-05
9	2.93E-04	2.77E-04	2.50E-04	4.95E-04	1.27E-03	1.02E-03	-1.45E-04
10	2.70E-04	6.36E-04	3.70E-04	6.26E-04	1.21E-03	1.04E-03	1.21E-05
11	2.36E-04	2.56E-04	2.38E-04	4.77E-04	1.14E-03	1.01E-03	-1.83E-04

Table S17. Values of Gi from the non-negative least square (NNLS) regression.

Samples	G2	G3	G4	G5	G6	G7	G10
1	2.32E-04	9.41E-04	2.13E-04	9.92E-04	1.37E-03	1.10E-03	4.57E-04
2	3.52E-04	2.21E-04	8.82E-05	6.55E-04	1.39E-03	1.22E-03	2.01E-04
3	3.00E-04	1.01E-03	3.42E-04	8.35E-04	1.27E-03	9.02E-04	2.48E-04
4	2.92E-04	4.40E-04	1.46E-04	7.04E-04	1.34E-03	1.16E-03	1.67E-04
5	3.74E-04	3.23E-04	2.01E-04	5.38E-04	1.33E-03	1.14E-03	0.00E+00
6	3.18E-04	3.36E-04	1.38E-04	4.84E-04	1.20E-03	1.05E-03	0.00E+00
7	2.71E-04	9.74E-04	3.75E-04	7.37E-04	1.22E-03	8.90E-04	1.06E-04
8	2.32E-04	3.71E-04	1.93E-04	7.36E-04	1.26E-03	1.14E-03	2.05E-05
9	3.14E-04	3.25E-04	8.38E-05	4.12E-04	1.27E-03	9.68E-04	0.00E+00
10	2.70E-04	6.36E-04	3.70E-04	6.26E-04	1.21E-03	1.04E-03	1.21E-05
11	2.62E-04	3.17E-04	2.87E-05	3.72E-04	1.14E-03	9.50E-04	0.00E+00

The **Gi** values are used for performing the individual regression to obtain the R square of the scatter plot of the initial dehydration rate against the concentration of OCFGs (Fig. 3d).

Table S18. Comparison of R^2 between OLS regression and NNLS regression.

Samples	R^2 of NNLS	R^2 of OLS
1	0.855	0.855
2	0.810	0.810
3	0.818	0.818
4	0.815	0.815
5	0.783	0.783
6	0.761	0.761
7	0.785	0.785
8	0.805	0.805
9	0.770	0.776
10	0.791	0.791
11	0.789	0.799

Table S19. Standardized values of Gi (Table S17) and dehydration rate by z-score normalization.

Samples	rate	G2	G3	G4	G5	G6	G7	G10
1	1.54E+00	-1.40E+00	1.41E+00	1.31E-01	1.97E+00	1.36E+00	4.51E-01	2.47E+00
2	-9.17E-01	1.38E+00	-1.10E+00	-9.67E-01	5.65E-02	1.53E+00	1.61E+00	6.47E-01
3	1.23E+00	1.80E-01	1.66E+00	1.26E+00	1.08E+00	-3.88E-03	-1.41E+00	9.80E-01
4	3.44E-01	-1.38E-02	-3.35E-01	-4.58E-01	3.35E-01	8.81E-01	1.02E+00	4.09E-01
5	-8.96E-01	1.88E+00	-7.44E-01	2.42E-02	-6.02E-01	7.38E-01	8.53E-01	-7.84E-01
6	-1.04E+00	5.88E-01	-6.96E-01	-5.28E-01	-9.11E-01	-1.00E+00	3.47E-03	-7.84E-01
7	1.20E+00	-4.96E-01	1.53E+00	1.56E+00	5.23E-01	-7.64E-01	-1.52E+00	-3.23E-02
8	-1.50E-01	-1.39E+00	-5.74E-01	-4.22E-02	5.19E-01	-1.29E-01	7.87E-01	-6.38E-01
9	-1.05E+00	4.98E-01	-7.35E-01	-1.01E+00	-1.32E+00	9.34E-03	-7.87E-01	-7.84E-01
10	7.81E-01	-5.18E-01	3.49E-01	1.52E+00	-1.08E-01	-8.27E-01	-6.91E-02	-6.98E-01
11	-1.05E+00	-7.03E-01	-7.64E-01	-1.49E+00	-1.54E+00	-1.79E+00	-9.51E-01	-7.84E-01

The data in Table S19 was standardized based on the values from the Table S17 (solved by the NNLS regression).

Figure S8

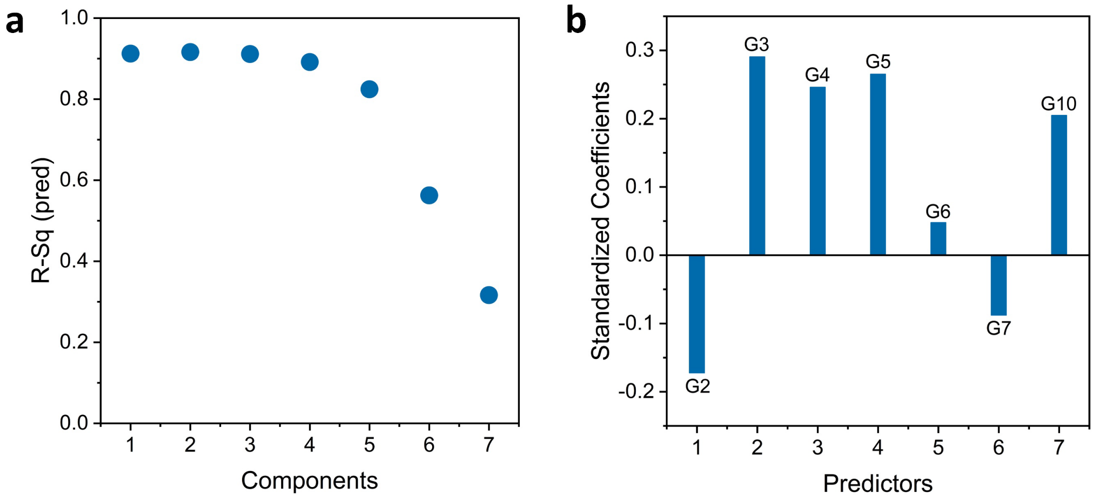

Figure S8. Partial least square (PLS) results. a PLS model selection plot from Leave-One-Out method. The R^2 (pred) values are optimal and comparable when using one to three components, so we elect to use a single component to mitigate overfitting concerns. Thus, the optimal model includes just the first PLS components. **b** Standardized coefficients of **Gi** from the PLS regression.

The Partial least square (PLS) analysis was performed using the data in Table S19.

Figure S9

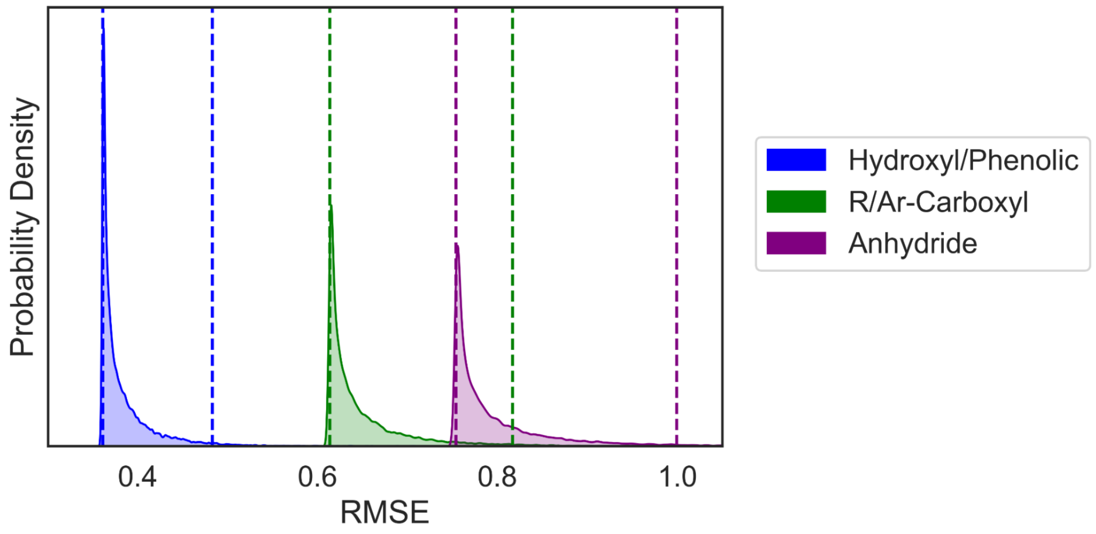

Figure S9. Bayesian Analysis.

To perform Bayesian inference, we generate a Markov chain Monte Carlo estimation of the posterior using the No-U-Turn Sampler of the Stan software.[11,12] Specifically, we obtain 20,000 samples from the posterior distribution of the linear regression relating measured reaction rates with each of our active site candidate's concentrations. For each of these 20,000 sampled regressions, we evaluate the root-mean-squared error (RMSE) and plot the resulting RMSE distributions in Figure S9 for three of the acidic active site candidates. With the 95% credible intervals of the hydroxyl/phenolic site showing the lowest RMSE range and not overlapping with any alternative candidates, this site offers the best linear regression with the measured reaction rates, and we can statistically conclude that this is the best active site candidate.

The Bayesian analysis was performed using the data in Table S19.

Figure S10

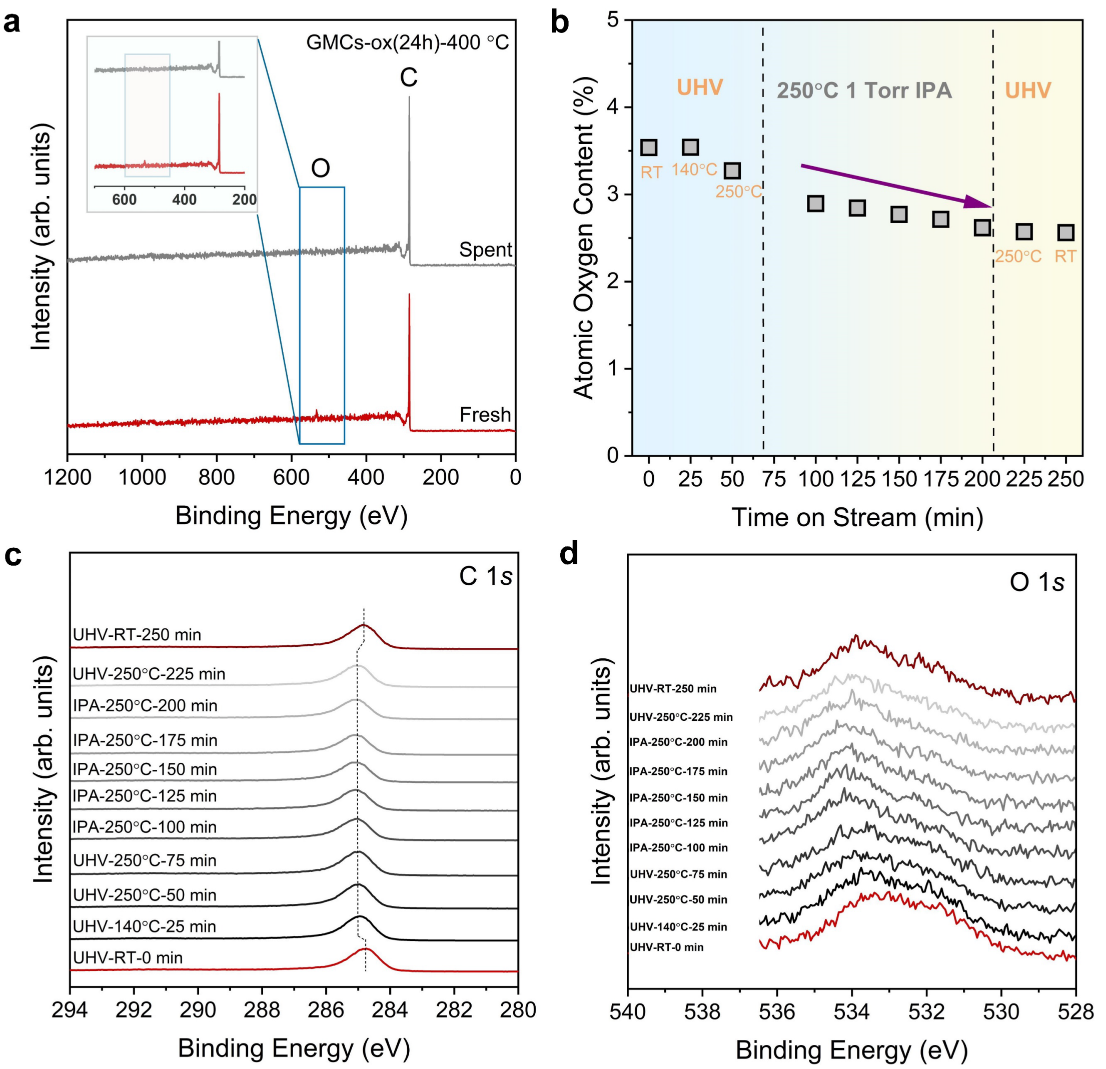

Figure S10. In situ XPS of GMCs-ox(24h)-400 ºC at different treatment conditions (UHV-250 °C before reaction, 1 torr IPA at 250 °C, UHV-250 °C after reaction, UHV-room temperature). a XPS survey scans of the fresh and spent samples. **b** atomic oxygen content with the spectra scans every 25 min **c** C 1*s* core-level region with time on stream at different treatments. The spectra were collected every 25 min. **d** O 1*s* core-level spectra with time on stream at different treatments. The spectra were collected every 25 min.

The oxygen content decreased with time on stream when the samples were heated to 250 °C, suggesting the thermal decomposition of the functional groups (e.g., carboxylic, or phenolic/hydroxyl groups).

Figure S11

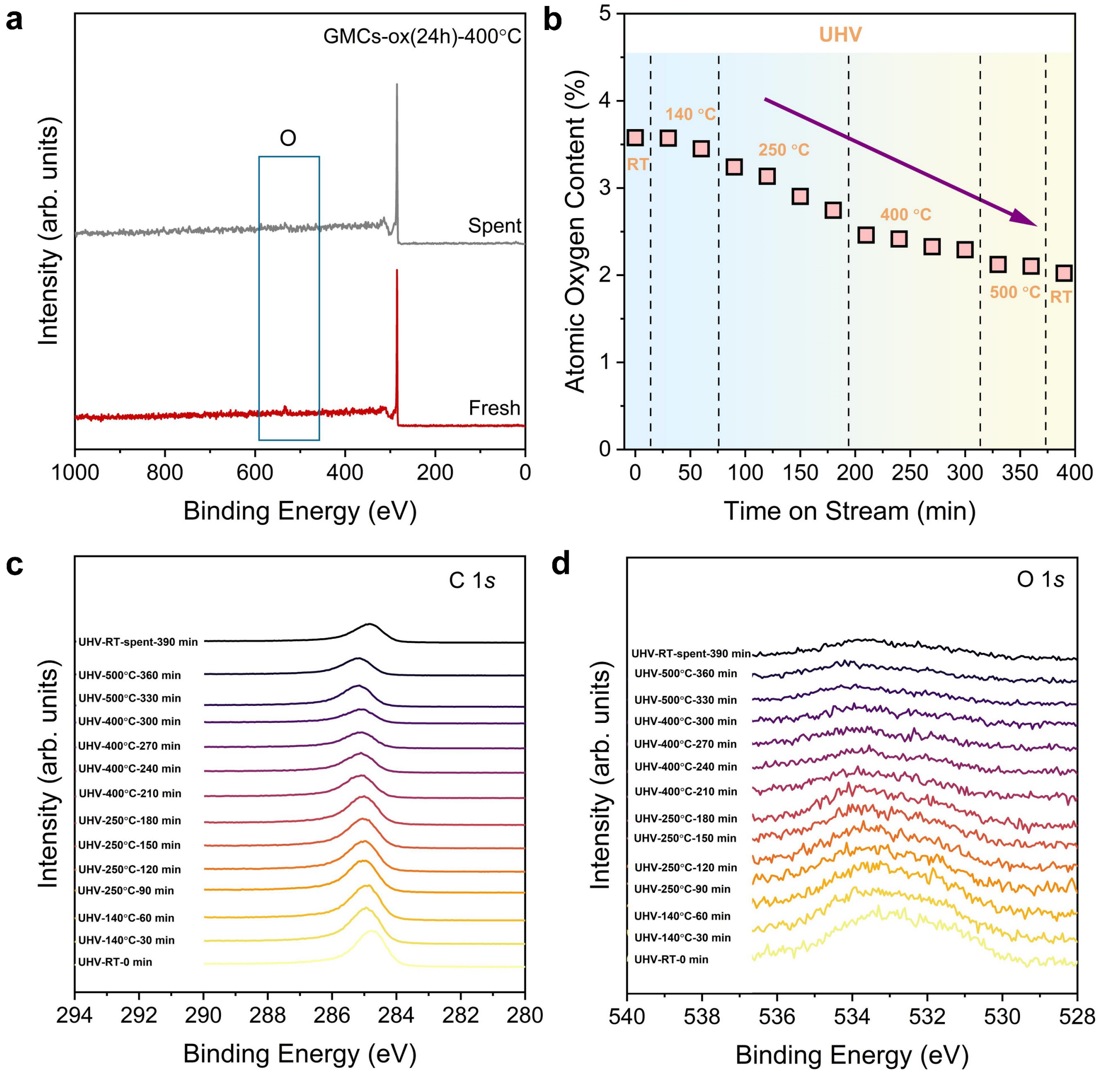

Figure S11. In situ XPS of GMCs-ox(24h)-400 °C with thermal stability measurements. a XPS survey scans of the fresh and spent samples. **b** atomic oxygen content under different annealing temperatures with the spectra scan every 30 min. **c** C 1*s* core-level region at different treatment temperatures (room temperature, 140 °C, 250 °C, 400 °C, 500 °C). The spectra were collected every 30 min. **d** O 1*s* core-level spectra with different annealing temperatures. Spectra were collected every 30 min.

The oxygen content decreased with time on stream when the samples were heated to 250 °C, suggesting the thermal decomposition of the functional groups (e.g., carboxylic, or phenolic/hydroxyl groups). The decreasing rate of the oxygen content in UHV is faster than in IPA, indicating that IPA was unavoidably adsorbed on carbon surfaces during the reaction.

Figure S12

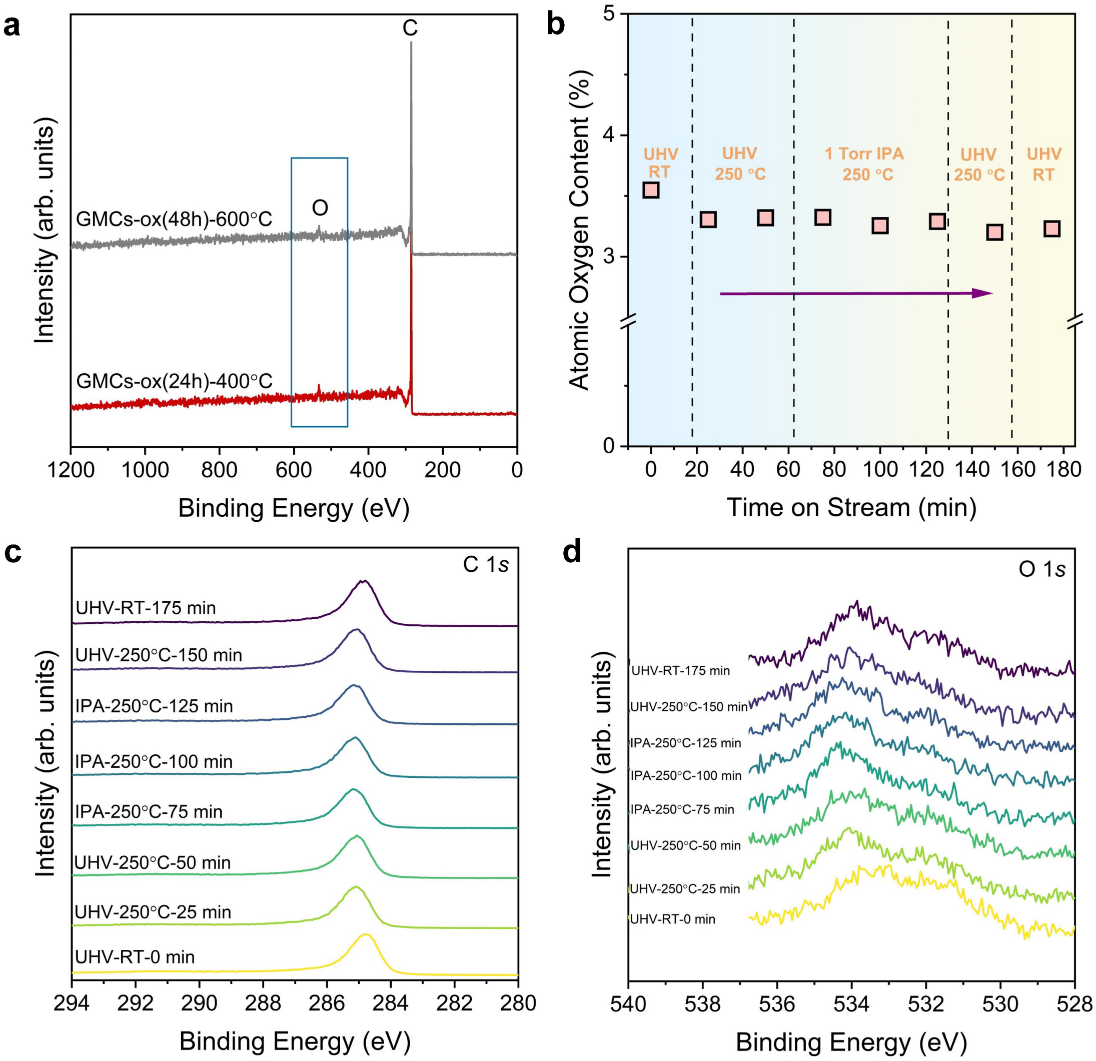

Figure S12. In situ XPS of GMCs-ox(48h)-600 ºC at different treatment conditions (UHV-250 °C before reaction, 1 torr IPA at 250 °C, UHV-250 °C after reaction, UHV at room temperature). a XPS survey scans of the fresh and spent samples. **b** atomic oxygen content with the spectra scans every 25 min. **c** C 1*s* core-level region with time on stream collected every 25 min. **d** O 1*s* core-level spectra with time on stream collected every 25 min.

Compared with GMCs-ox(24h)-400 °C, GMCs-ox(48h)-600 °C have identical oxygen content but different reactivity and thermal stability. However, when exposed to IPA, the oxygen content remains unchanged, indicating no reaction on the sample surfaces. Furthermore, OCFGs are thermally stable at 250 °C during the reaction due to low concentrations of -OH and -COOH on the sample surfaces, consistent with the IPA dehydration (Figure S13) and TPDE-MS results (Fig. 2a).

Figure S13

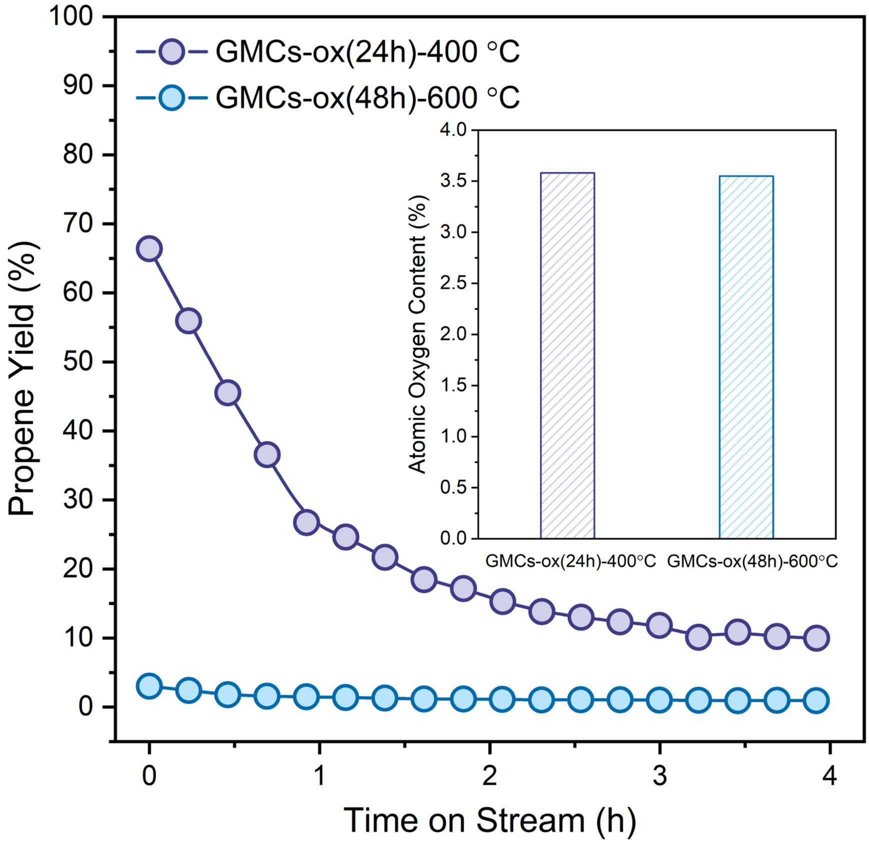

Figure S13. Reaction of 2-proponal over GMCs-ox(24h)-400 ºC and GMCs-ox(48h)-600 ºC (inset: atomic oxygen content of the two samples). Reaction conditions: 4 mol% 2-propanol, 100 mL/min N_2/2-propanol mixture, 50 mg catalyst, reaction temperature of 250 °C. Inset: atomic oxygen content of the fresh catalysts.

Figure S13 depicts the 2-propanol dehydration over samples with identical oxygen content but different acidic OCFGs distributions. Clearly, the propene yield is low on GMCs-ox(48h)-600 ºC due to low -OH and -COOH concentration.

Figure S14

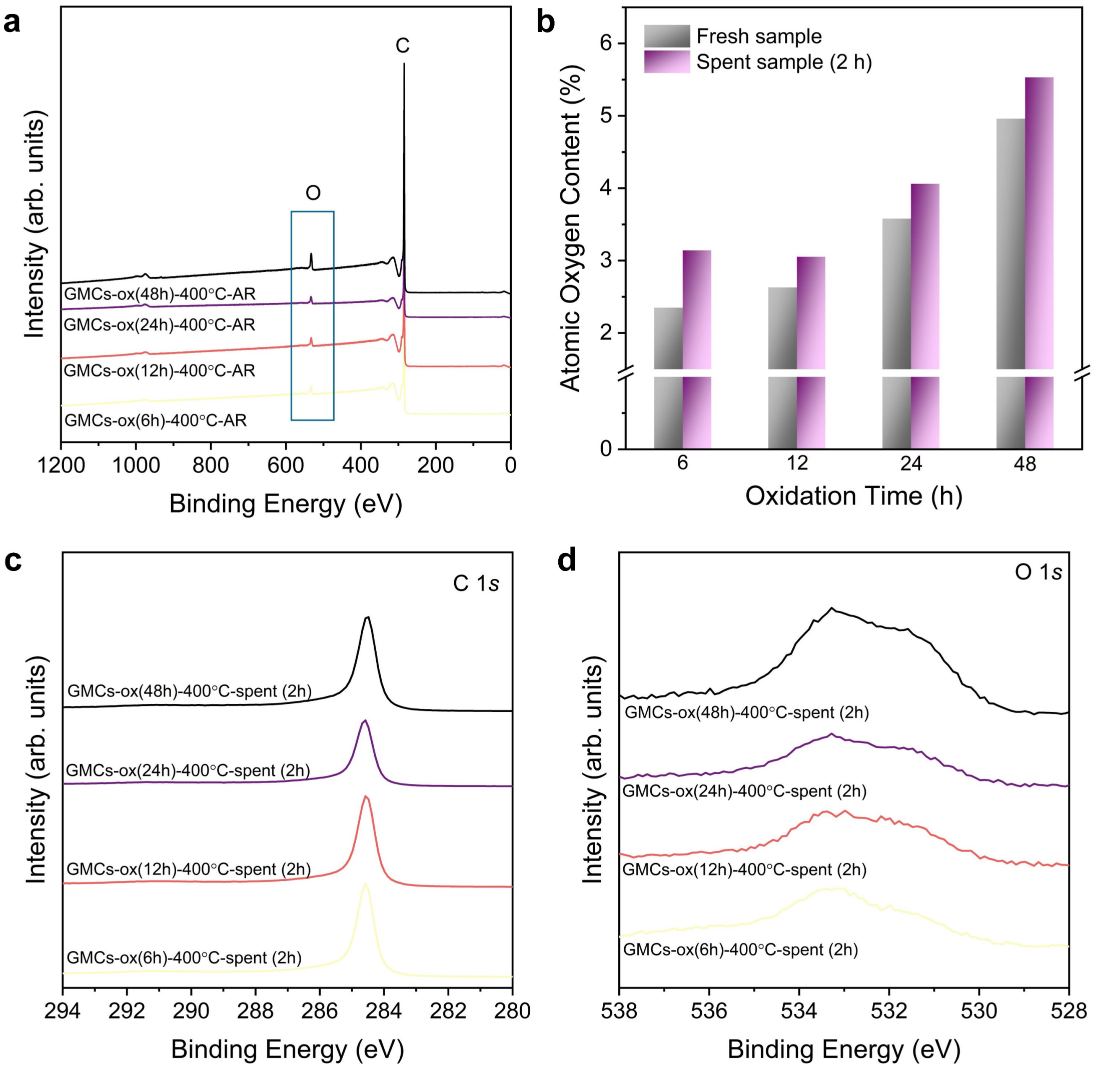

Figure S14. Ex situ XPS of spent (TOS = 2 h) GMCs-ox (*a* h)-400 °C samples with different oxidation times (*a* = 6, 12, 24, 48). **a** XPS survey scans of the spent samples. **b** atomic oxygen content of fresh and spent catalysts. **c** C 1*s* core-level spectra. **d** O 1*s* core-level spectra. Reaction conditions: 4 mol% 2-propanol, 100 mL/min N_2/2-propanol mixture, 50 mg catalyst, reaction temperature = 250 °C.

The increasing oxygen content on spent catalysts suggests adsorption of reactants and/or products on the carbon surfaces.

Figure S15

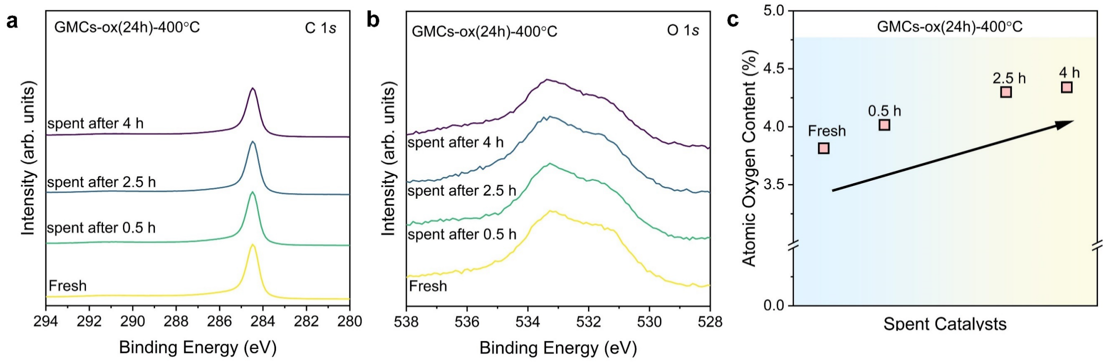

Figure S15. Ex situ XPS of spent GMCs-ox (24 h)-400 ºC samples (TOS = 0, 0.5 h, 2.5 h, 4 h). **a** C 1*s* core-level spectra, **b** O 1*s* core-level spectra. **c** atomic oxygen content. Reaction conditions: 4 mol% 2-propanol, 100 mL/min N_2/2-propanol mixture, 50 mg catalyst, reaction temperature = 250 °C.

It is challenging to analyze the dynamic change of OCFGs of the spent catalysts during reactions due to following reasons: (1) The reaction rate cannot be monitored on the NAP-XPS instrument. (2) Ex situ XPS measurement of the spent samples cannot prevent complete contamination from air even with the air-free vacuum transfer module. As shown in Figure S15c, the oxygen content increases with time-on-stream, suggesting H_2O and alcohol originating from the reactant and products or some contamination from air adsorbed on the spent catalysts.

Figure S16

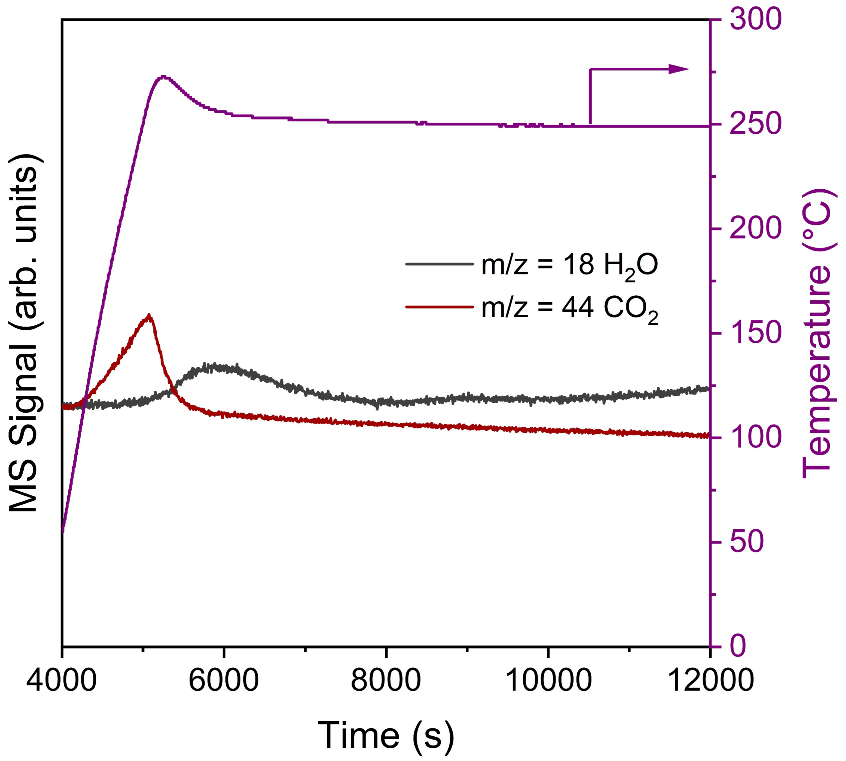

Figure S16. TPDE-MS profiles of GMCs-ox(24h)-400 °C in He at 250 ºC with a heating rate of 10 °C/min.

Figure S17

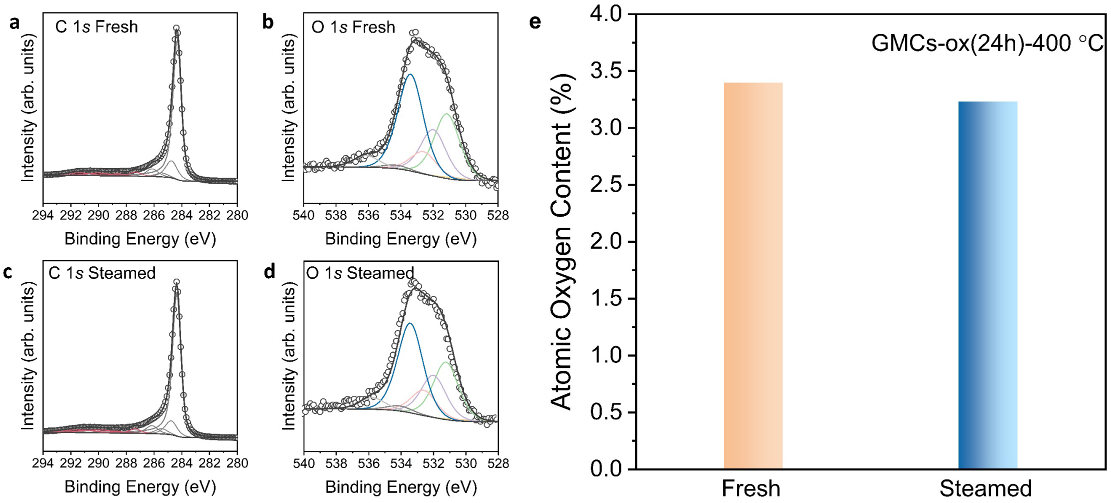

Figure S17. Air free XPS data. a, **b** C 1*s*, O 1*s* fitting of fresh GMCs-ox(24h)-400 °C sample. **c**, **d** C 1*s*, O 1*s* fitting of steamed GMCs-ox(24h)-400 °C sample. Treatment conditions: 10 mol% steam, 100 mL/min N_2/Steam mixture, 50 mg catalyst, reaction temperature of 250 °C, **e** Atomic oxygen content of fresh and steamed sample.

Compare the fresh sample with the steamed one, the oxygen content and the distribution of the OCFGs are almost the same, indicating that the in situ formed carboxylic groups (from hydrolysis of carboxylic anhydride by steaming in 0.1 bar H_2O at 250 °C) compensate for the thermal decomposition of the functional groups (Fig. 3e).

Supplementary Note 4

Estimation of TOF of active centers

The number of -OH (phenolic/hydroxyl groups) and -COOH (carboxylic groups) are estimated from ex situ C 1*s* and O 1*s* spectra deconvolution and the **Gi** calculated by data analysis (see Methods, Supplementary Note 3). Specifically, the oxygen content of the surface is comparable to the bulk due to the distribution of oxygen atoms is perfectly uniform in the depth direction (Fig. S6)

To validate that -OH is the dominant active site, we estimated the TOFs on the active centers from the statistical perspective. We use one 2-parameter model (A), supported by our data and three 1-parameter models by assuming that (1) all exposed -OH and -COOH are on the outer surfaces of carbon (Table S2) (for model A, B, C, D), (2) all exposed -OH and -COOH are equally active sites (model B), (3) only -OH is active for alcohol dehydration (model C), or (4) only -COOH is active (model D).

Model A $$R^{DH} = TOF^{DH}_{\text{-}OH} \times n^{DH}_{\text{-}OH} + TOF^{DH}_{\text{-}COOH} \times n^{DH}_{\text{-}COOH}$$

Model B $$R^{DH} = TOF^{DH}_{outer\ surface} \times (n^{DH}_{\text{-}OH} + n^{DH}_{\text{-}COOH})$$

Model C $$R^{DH} = TOF^{DH}_{\text{-}OH} \times n^{DH}_{\text{-}OH}$$

Model D $$R^{DH} = TOF^{DH}_{\text{-}COOH} \times n^{DH}_{\text{-}COOH}$$

Model D shows the lowest Adj. R^2 value, suggesting that -COOH alone cannot fully capture the variability of the rate. In contrast, Model C shows a higher Adj. R^2 value, indicating that -OH is a better active site candidate than -COOH. Besides, the Adj.R^2 values of Model A and Model B are slightly lower than those of Model C due to the overfitting after adding the -COOH term, further implying the poor correlation between -COOH and dehydration rate. More importantly, the confidence interval (Table S20) of -COOH in Model A is between -0.509 and 0.573 which includes 0, indicating the possibility of zero-TOF of -COOH. In other words, compared to -OH, -COOH has a negligible contribution to dehydration activity. Overall, the TOF fitting results based on the 4 models demonstrate that -OH is the best active site for the alcohol dehydration reaction.

Table S20. TOFs and statistics for various kinetic models.

Models	TOF (s^{-1})			Statistics	
	-OH	-COOH	Surface protonated OCFGs	RSS*	Adj.R^2
Model A	0.225	0.032	n/a	2.90E-08	0.7628
Model B	n/a	n/a	0.173	3.02E-08	0.7821
Model C	0.236	n/a	n/a	2.91E-08	0.7863
Model D	n/a	0.600	n/a	4.92E-08	0.7076

RSS*=The residual sum of squares

Table S21. Confidence interval of TOFs with different models.

Models	Confidence interval (lower 95%, upper 95%)		
	-OH	-COOH	Surface protonated OCFGs
Model A	(0.022, 0.428)	(-0.509, 0.573)	n/a
Model B	n/a	n/a	(0.128, 0.218)
Model C	(0.177, 0.296)	n/a	n/a
Model D	n/a	(0.394, 0.807)	n/a

Figure S18

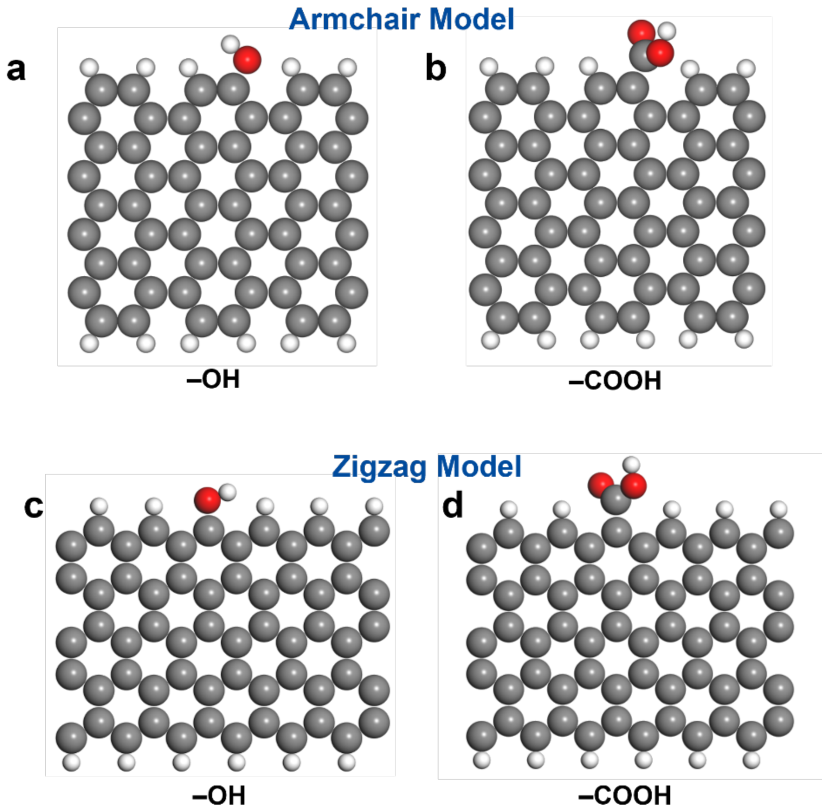

Figure S18. Structures for DFT modeling. a -OH, and **b** -COOH on the graphene ribbons with armchair edges; **c** -OH, and **d** -COOH on the graphene ribbons with zigzag edges.

Figure S19

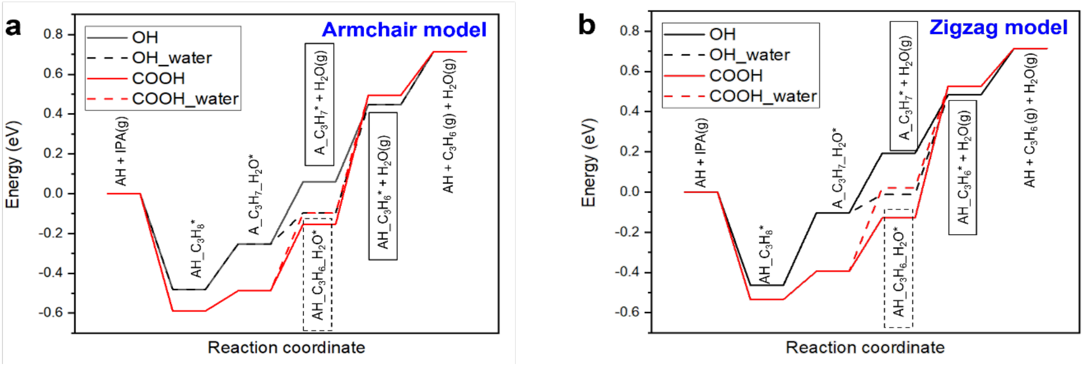

Figure S19. Energy profiles of IPA dehydration over -OH and -COOH. a armchair-edge model. **b** zigzag-edge model.

Figure S20

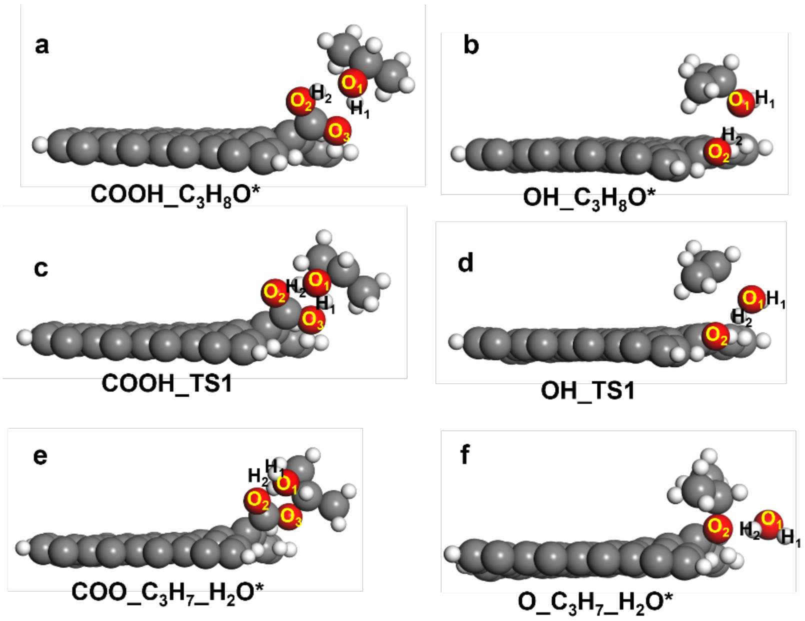

Figure S20. Configurations of C_3H_8O (intermediates) on **a** -COOH and **b** -OH sites; configurations of transition states associated with the concerted proton transfer and C-O bond cleavage (first transition state) on **c** -COOH and **d** -OH sites; configurations of $C_3H_7O\_H_2O^*$ (intermediates) on **e** -COOH and **f** -OH sites.

Figure S21

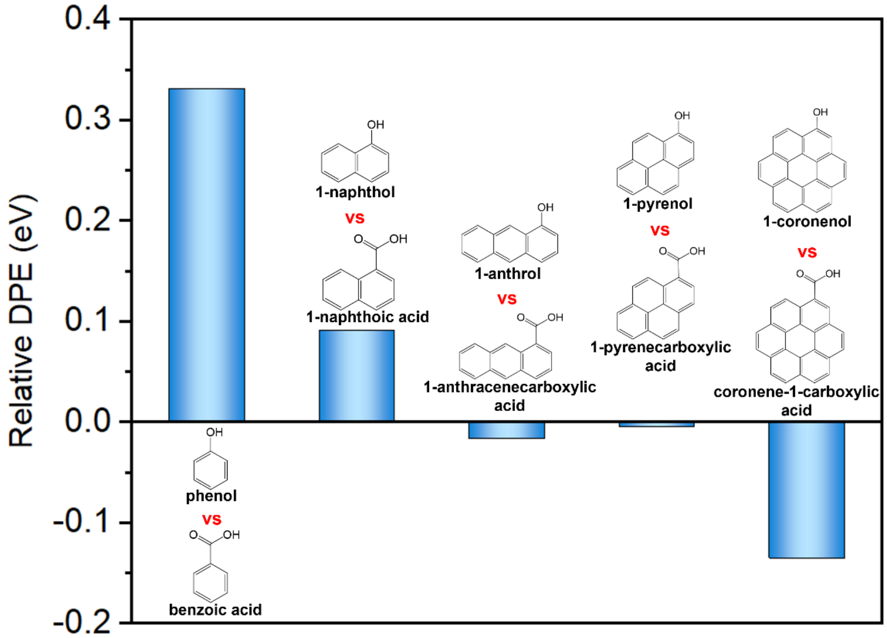

Figure S21. Relative DPE (DPE_{-OH}-DPE_{-COOH}) for aromatic alcohols and acids with varying number of fused aromatic rings: phenol, benzoic acid; 1-naphthol, 1-naphthoic acid; 1-anthrol, 1-anthracenecarboxylic acid; 1-pyrenol, 1-pyrenecarboxylic acid; and 1-coronenol, coronene-1-carboxylic acid.

Figure S22

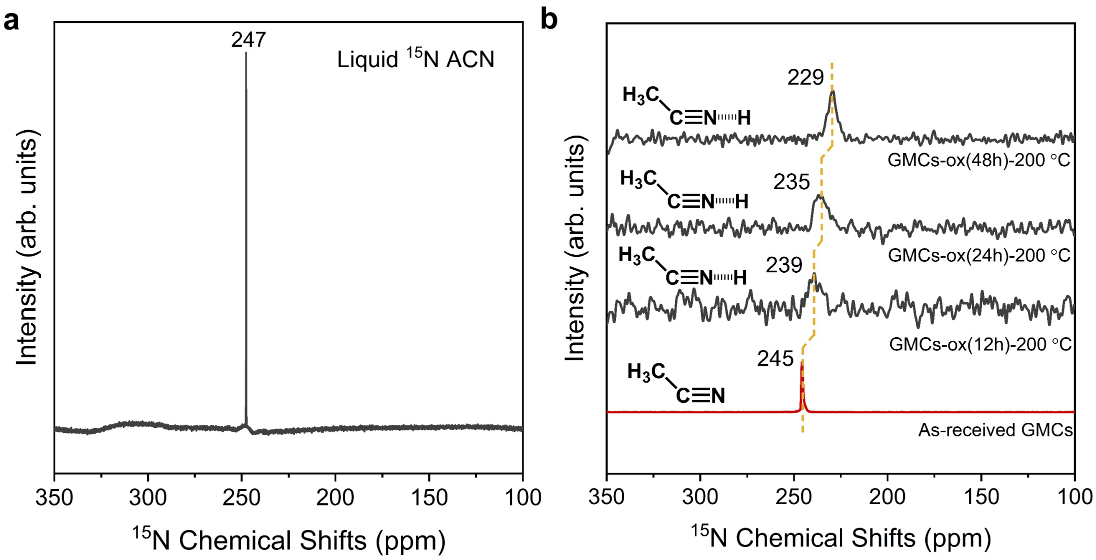

Figure S22. NMR spectra for acid site quantification. a ^{15}N NMR spectra for the ^{15}N acetonitrile, **b** ^{15}N MAS NMR spectra of acetonitrile adsorbed on oxygenated carbon. Samples were treated with gas-phase ^{15}N ACN.

Supplementary Note 5

Liquid-absorbed ^{15}N ACN NMR

Figure S23

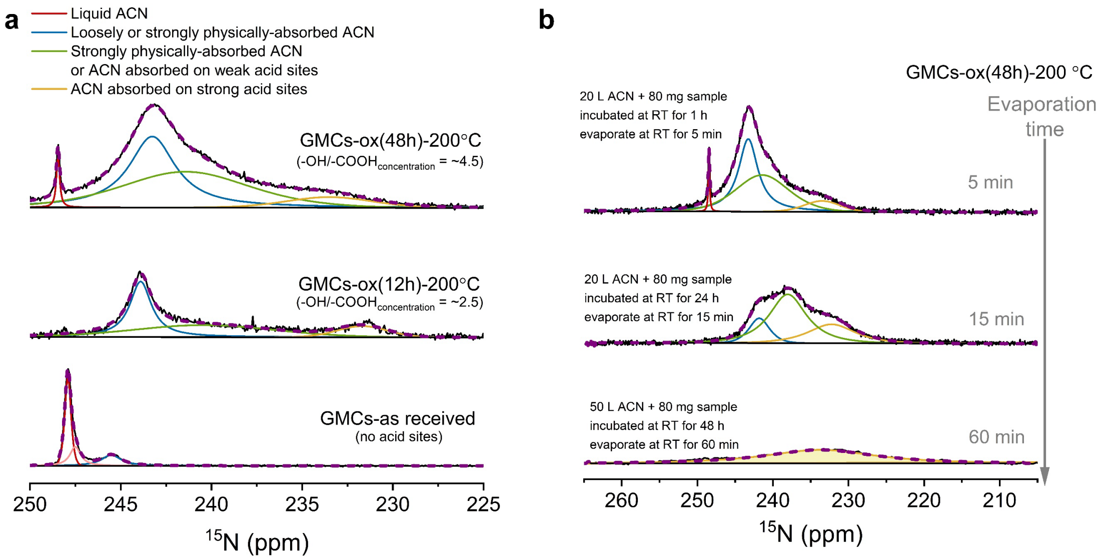

Figure S23. NMR spectra for acid site quantification. a ^{15}N MAS NMR spectra of acetonitrile adsorbed on oxygenated carbon. **b** ^{15}N MAS NMR spectra of acetonitrile adsorbed on GMCs-ox(48h)-200 °C; the spectra were collected with different evaporation times. Samples were treated by liquid phase ^{15}N ACN.

We further conducted the NMR with the loadings above monolayer (the samples treated with liquid ^{15}N ACN) (Fig. S23 a, b) to distinguish the acid sites with different acidity. In Fig. S23b, with the increase of the evaporation time, the peaks disappear with the order of the ACN desorption rate. There is only one broad peak left after 60 min evaporation. Therefore, the assignments of the peaks can be identified by the combination of ACN desorption rate and our DFT calculations (Fig. 4f). The four peaks are *i*) pseudo-liquid ACN, *ii*) physically absorbed ACN, *iii*) strongly physically absorbed ACN & ACN absorbed on weak acid sites (most probably the -OH associated with the alkyl rings). *iv*) ACN absorbed on strong protonated acid sites (including -OH/-COOH associated with different numbers of benzene rings), respectively. The very broad last peak suggests that the strong acid sites are overlapped due to the complex carbon microenvironment.

To resolve the overlapping of the acid sites of the last broad peak in Fig S23b, the measurement at cryogenic temperatures (-25 °C ~ -75 °C to freeze ACN) is performed. In Fig. S24, the peak width is gradually narrower with the decreasing of the measurement temperature, however, no more peaks were observed.

We concluded that the peaks assigned to the strong acid sites should be the overlapping of the different acidic protonated OCFGs, including -COOH associated with alkyl rings or benzene rings but with fewer ring numbers or -OH associated with a greater number of benzene rings (phenolic groups).

Figure S24

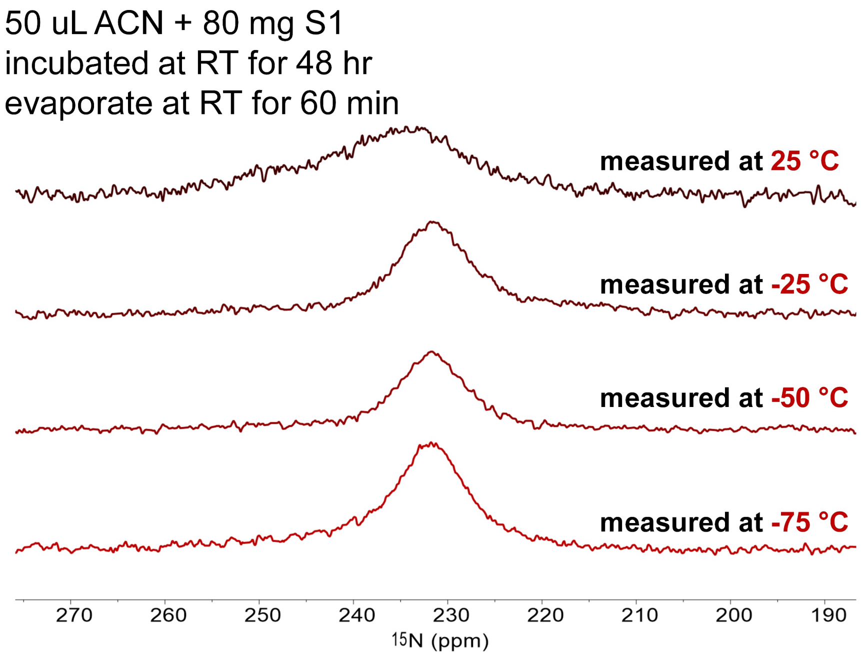

Figure S24. NMR spectra for acid site quantification. ^{15}N MAS NMR spectra of acetonitrile adsorbed on GMCs-ox(48h)-200 °C after 60 min evaporation, the spectra measured at different temperatures. Samples were treated by liquid phase ^{15}N ACN.

Supplementary References

1 Kundu, S., Wang, Y., Xia, W. & Muhler, M. Thermal stability and reducibility of oxygen-containing functional groups on multiwalled carbon nanotube surfaces: a quantitative high-resolution XPS and TPD/TPR study. *J. Phys. Chem. C* **112**, 16869-16878 (2008).

2 Dutta, S. et al. Solventless C-C coupling of low carbon furanics to high carbon fuel precursors using an improved graphene oxide carbocatalyst. *ACS Catal.* **7**, 3905-3915 (2017).

3 Zielke, U., Hüttinger, K. & Hoffman, W. Surface-oxidized carbon fibers: I. Surface structure and chemistry. *Carbon* **34**, 983-998 (1996).

4 Zhou, J. H. et al. Characterization of surface oxygen complexes on carbon nanofibers by TPD, XPS and FTIR. *Carbon* **45**, 785-796 (2007).

5 Schuster, M. E. et al. Surface sensitive study to determine the reactivity of soot with the focus on the European emission standards IV and VI. *J. Phys. Chem. A* **115**, 2568-2580 (2011).

6 Blume, R. et al. Characterizing graphitic carbon with X-ray photoelectron spectroscopy: A step-by-step approach. *ChemCatChem* **7**, 2871 (2015).

7 Gengenbach, T. R., Major, G. H., Linford, M. R. & Easton, C. D. Practical guides for x-ray photoelectron spectroscopy (XPS): Interpreting the carbon 1s spectrum. *J. Vac. Sci. Technol. A* **39**, 013204 (2021).

8 Reiche, S. et al. Reactivity of mesoporous carbon against water-An in-situ XPS study. *Carbon* **77**, 175-183 (2014).

9 Friedel Ortega, K., Arrigo, R., Frank, B., Schlögl, R. & Trunschke, A. Acid–base properties of N-doped carbon nanotubes: a combined temperature-programmed desorption, X-ray photoelectron spectroscopy, and 2-propanol reaction investigation. *Chem. Mater.* **28**, 6826-6839 (2016).

10 Skoog, Douglas A., F. James Holler, and Stanley R. Crouch. Principles of instrumental analysis. Cengage learning, 2017.

11 Hoffman, M. D. & Gelman, A. The No-U-Turn sampler: adaptively setting path lengths in hamiltonian monte carlo. *J. Mach. Learn. Res.* **15**, 1593-1623 (2014).

12 Carpenter, B. et al. Stan: A probabilistic programming language. *J. Stat. Softw.* **76**, (2017).

13 Wertheim, G. & Citrin, P. Fermi surface excitations in X-ray photoemission line shapes from metals. Photoemission in solids I, 197-236 (1978).
