# Supplementary figures and images for: Tuning the reactivity of carbon surfaces with oxygen-containing functional groups
Source: Nat Commun. 2023 Apr 21;14:2293. doi: 10.1038/s41467-023-37962-3 (PMC10121666; doi:10.1038/s41467-023-37962-3)

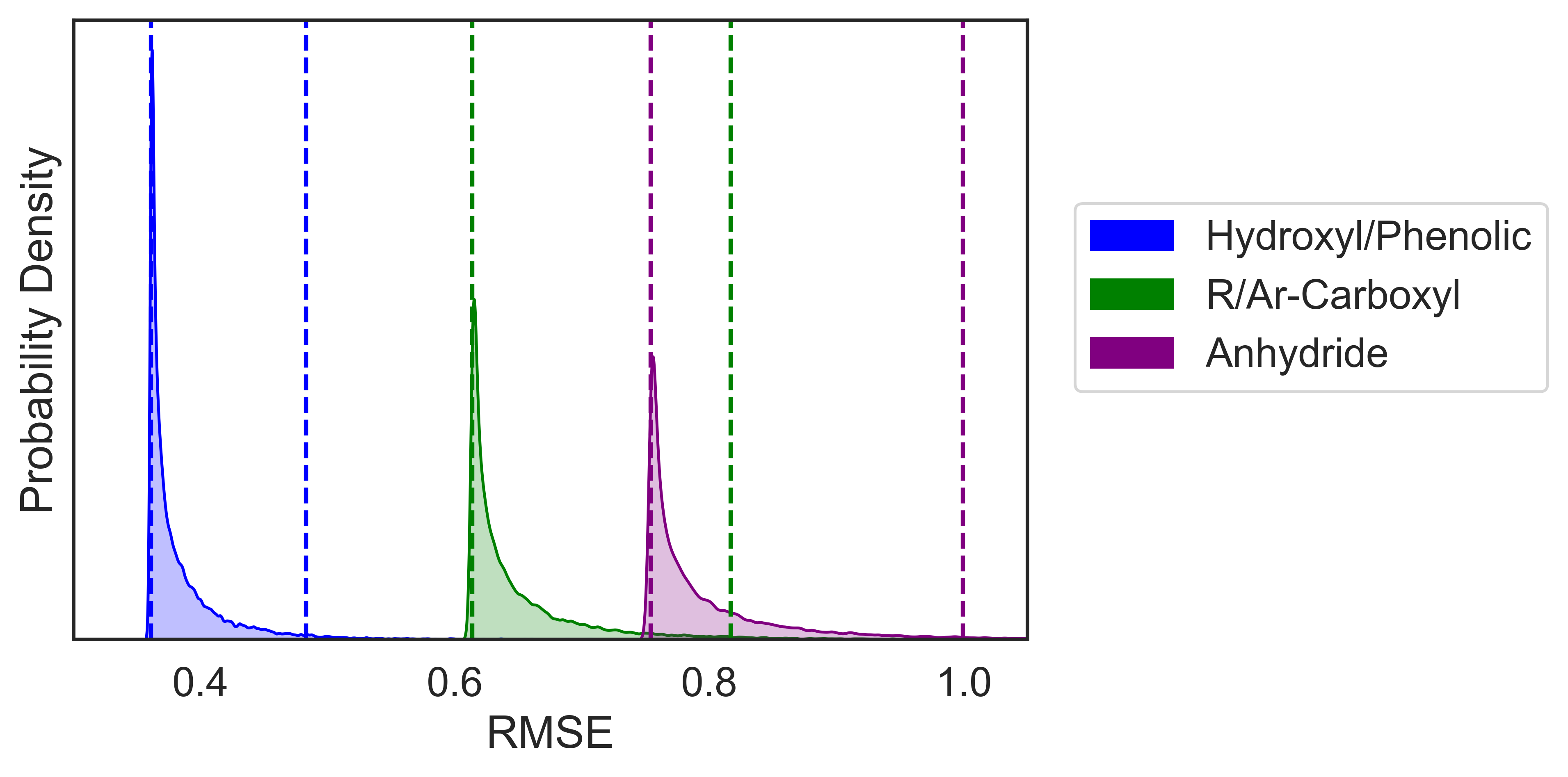

Supplement: Supplementary file 3 — Source Data [file 41467_2023_37962_MOESM3_ESM.zip › Dataset for tuning oxygen-containing functional groups and reactivity of carbon surfaces-update/Bayesian Analysis/RMSE.png]
